# Metabolomic insights of macrophage responses to graphene nanoplatelets: Role of scavenger receptor CD36

**DOI:** 10.1371/journal.pone.0207042

**Published:** 2018-11-07

**Authors:** Sherleen Xue-Fu Adamson, Ruoxing Wang, Wenzhuo Wu, Bruce Cooper, Jonathan Shannahan

**Affiliations:** 1 School of Health Sciences, Purdue University, West Lafayette, IN, United States of America; 2 School of Industrial Engineering, Purdue University, West Lafayette, IN, United States of America; 3 Metabolite Profiling Facility in Bindley Biosciences Center, Discovery Park, Purdue University, West Lafayette, IN, United States of America; University of South Alabama Mitchell Cancer Institute, UNITED STATES

## Abstract

Graphene nanoplatelets (GNPs) are novel two-dimensional engineered nanomaterials consisting of planar stacks of graphene. Although human exposures are increasing, our knowledge is lacking regarding immune-specific responses to GNPs and mechanisms of interactions. Our current study utilizes a metabolite profiling approach to evaluate macrophage responses to GNPs. Furthermore, we assessed the role of the scavenger receptor CD36 in mediating these GNP-induced responses. GNPs were purchased with dimensions of 2 μm × 2 μm × 12 nm. Macrophages were exposed to GNPs at different concentrations of 0, 25, 50, or 100 μg/ml for 1, 3, or 6 h. Following exposure, no cytotoxicity was observed, while GNPs readily associated with macrophages in a concentration-dependent manner. After the 1h-pretreatment of either a CD36 competitive ligand sulfo-N-succinimidyl oleate (SSO) or a CD36 specific antibody, the cellular association of GNPs by macrophages was significantly reduced. GNP exposure was determined to alter mitochondrial membrane potential while the pretreatment with a CD36 antibody inhibited these changes. In a separate exposure, macrophages were exposed to GNPs at concentrations of 0, 50, or 100 μg/mL for 1 or 3h or 100 μM SSO (a CD36 specific ligand) for 1h and collected for metabolite profiling. Principal component analysis of identified compounds determined differential grouping based on exposure conditions. The number of compounds changed following exposure was determined to be both concentration- and time-dependent. Identified metabolites were determined to relate to several metabolism pathways such as glutathione metabolism, Pantothenate and CoA biosynthesis, Sphingolipid metabolism, Purine metabolism, arachidonic acid metabolism and others. Lastly, a number of metabolites were found in common between cells exposed to the CD36 receptor ligand, SSO, and GNPs suggesting both CD36-dependent and independent responses to GNP exposure. Together our data demonstrates GNP-macrophage interactions, the role of CD36 in the cellular response, and metabolic pathways disrupted due to exposure.

## Introduction

Engineered nanomaterials (ENMs) have markedly revolutionized numerous technology fields due to their novel and diverse physicochemical properties. Graphene nanoplates (GNPs), a derivative from graphene, have a unique two-dimensional (2D) sheet structure consisting of a small planar stack of graphene layers with an average thickness within the nanorange but with length and width dimensions ranging up to microns. This structure allows GNPs to have properties consisting of uniform shape, high surface area to weigh ratios, high conductivity of electricity and heat, ability to undergo a variety of surface modifications, and flexibility [1, 2]. In comparison to single layer graphene, GNPs are cheaper to manufacture and have enhanced barrier and mechanical properties (stiffness, strength, and surface hardness). Therefore GNPs have been widely applicable in the development of nanoelectronics, energy storage, solar cells, current collector, biosensor, drug delivery, biomedicine, and phototherapies of cancer [1–4]. Concurrently, the rapid increase in GNP production and applications could increase the risk of unintentional occupational and environmental exposure and have raised concerns regarding the potential toxic impacts of GNPs on human health [5–7].

Several recent reviews have provided a comprehensive overview regarding the potential toxicity associated with graphene-family nanoparticle exposures [8–10]. A number of studies have examined the *in vitro* cytotoxicity associated with the dose, time, morphology, functionalization, size, and surface coating of graphene in different cell types, as well as the *in vivo* toxicity through different exposure routes such as pulmonary, oral, intravenous, intraperitoneal, and intravitreal administrations [10–13]. However, few studies have been performed to specifically evaluate the toxicity induced by GNPs. Chang and colleagues observed significantly lower cytotoxicity in human lung carcinoma epithelial A549 cells following exposure to oxidized GNPs compared to graphene oxide nanoribbons[14]. When investigating the cytotoxicity of reduced oxide GNPs of differing sizes, the induced genotoxicity and DNA fragmentation were determined to be size-dependent in human mesenchymal stem cells [15]. Both *in vivo* and *in vitro* studies found that GNPs functionalized with biocompatible polymer dextran did not induce hematological toxicity [16]. Park et al. determined that GNPs were biopersistent within the lung remaining 28 and 90 days following a single instillation, while the secretion of inflammatory cytokines maximized on day 14 [17]. *In vitro* experiments using human bronchial epithelial cells (BEAS-2B) demonstrated concentration-dependent reduction in cell viability, intracellular encapsulated of GNPs in autophagosome-like vacuoles, down-regulation of reactive oxygen species, suppressed ATP production, mitochondrial damage, and elevated expressions of autophagy-related proteins [18]. Further studies, however, are necessary to explore the mechanisms of GNP-induced toxicity such as interactions with immune cells that are likely to occur following exposures.

Macrophages interact with particles through a wide variety of cell-surface receptors such as scavenger receptors (SRs), which facilitate phagocytosis and the immune response [19]. SRs have been shown to mediate the internalization of various nanoparticles (i.e., silver, silica, and titanium oxide nanoparticles, carbon nanotubes, etc) by macrophages [20–23]. CD36 (also named SR-B2), binds various ligands and is specifically is known to facilitate macrophage interactions with oxidized low-density lipoprotein (OxLDL) promoting foam cell formation, platelet activation/aggregation, apoptosis, angiogenesis, and inflammation [24–27]. The binding of ligands activates CD36 triggering diverse intracellular signaling events associated with energy production, mitochondria, apoptosis and oxidative stress, which could interfere the activation, migration and normal function of macrophages [26, 28–30]. However, how macrophages interact with GNPs and whether CD36 plays a role in mediating the uptake of GNPs and the immune responses have not been evaluated.

The rapid expansion of ENM use in a variety of engineering and biomedical applications has urged the need to understand potential toxicity. Advanced high-throughput methodologies such as multi-omics methodologies are particularly more suitable and efficient to globally assess the potential toxicity of ENMs than traditional analytical toxicology methods [31–33]. Metabolomics, specifically, is a powerful tool to identify endogenous metabolism molecules that may be modified in response to ENM insults thus providing mechanistic insights [32, 34–36]. Recent studies have employed a metabolomics approach to evaluate biological responses to different ENMs including copper oxide NPs, silver, titanium oxide, and gold nanoparticles, as well as graphene [37–43]. To date no investigation has been performed to specifically evaluate the global metabolome of immune cells, such macrophages, which will likely interact with GNPs following exposure.

**Fig 1:**
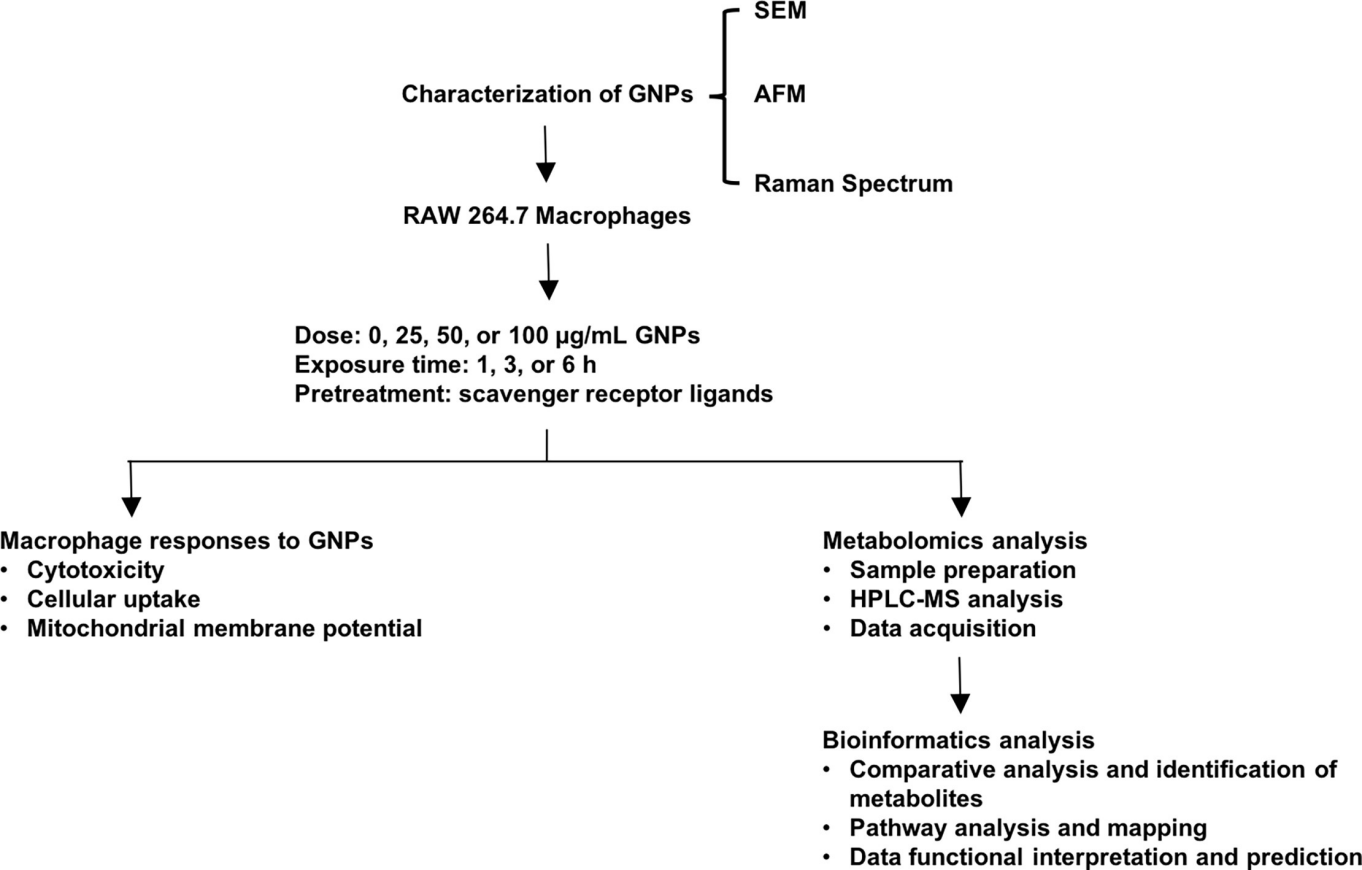
Flow chart of experiments to evaluate mechanisms of toxicity of GNPs in mouse macrophages: *in vitro*.

Therefore, the current study was designed to utilize a metabolite profiling approach to evaluate the macrophage responses to GNPs and to specifically assess the role of the scavenger receptor CD36 (Fig 1). GNPs without functionalization were characterized prior to *in vitro* experiments evaluating macrophage cytotoxicity, uptake, mitochondrial membrane potential and alterations in metabolite profiles. A subset of macrophages received treatment with either a CD36 specific ligand or antibody prior to GNP exposure to specifically examine the role of CD36 in GNP-induced macrophage responses. To the best of our knowledge, this is the first report describing GNP-induced alterations in macrophage metabolism and the role of CD36. Data from this study enhances our understanding of GNP-induced toxic effects, as well as provides metabolomic insight for future mechanistic assessment of ENMs.

## Materials and methods

### Graphene nanoplatelet characterization

The GNPs (Catalog No.: SKU 05017) were purchased from Cheap Tubes Inc. (Cambridgeport, VT). The morphologies of GNPs were characterized by a field emission scanning electron microscope (FESEM, Hitachi S-4800 Field Emission SEM). The thicknesses were determined by atomic force microscope (AFM, Keysight 5500). The Raman spectrum was carried out on a Horiba LabRAM HR800 Raman spectrometer at room temperature to confirm the composition using a He-Ne laser (wavelength 632.8 nm).

### Macrophage cell culture

RAW264.7 mouse macrophages were cultured in DMEM medium supplemented with 10% FBS and 100 U/mL penicillin-streptomycin. Macrophages were maintained in cell culture dishes under standard conditions at 37°C and 5% CO_2_. All GNP exposures and subsequent treatments were performed in serum free medium (SFM).

### Cell viability

**Fig 2:**
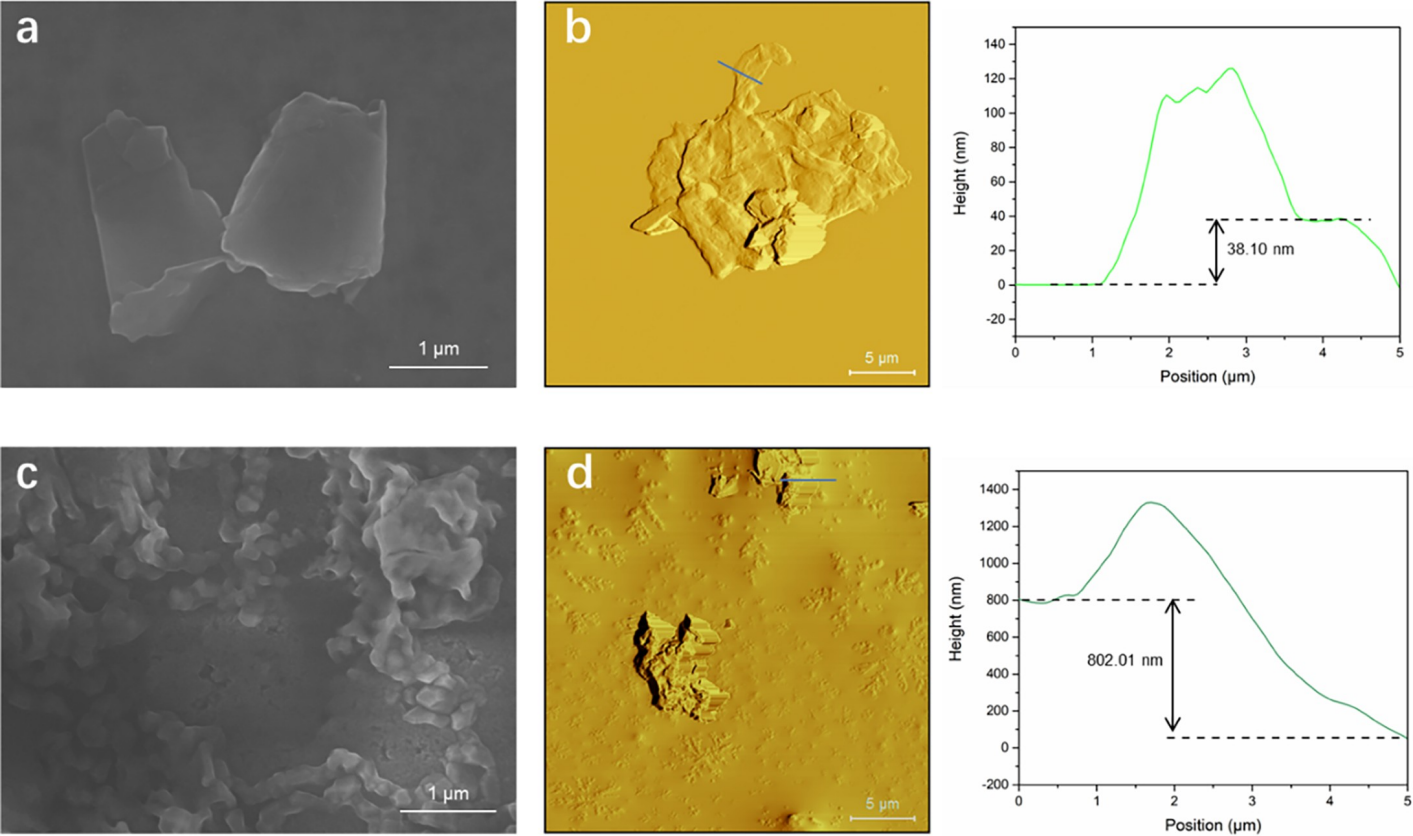
Characterization of GNPs dispersed in water and in serum free medium. (a) SEM image and (b) AFM image with corresponding line-scan profile of GNPs dispersed in water. (c) SEM image and (d) AFM image with corresponding line-scan profile of GNPs dispersed in SFM.

Macrophages were grown to 90% confluency in 24-well plates and were then exposed to 0, 25, 50, or 100 μg/mL GNPs, or 100 μg/mL zinc oxide (ZnO) NPs as the positive control for 1, 3, and 6 h in SFM. Changes in cell viability were evaluated using the propidium iodide nucleic acid staining assay (Invitrogen, Catalog number P3566, Carlsbad, CA) following manufacturer’s instructions and analyzed using the flow cytometry (Accuri C6 Flow Cytometer, BD Biosciences, San Jose, CA). No overt cytotoxicity was identified in all three selected GNPs concentrations (25, 50, or 100 μg/mL) across any of the time points therefore the concentrations of 50 and 100 μg/mL GNPs were utilized for all subsequent experiments (Fig 2A).

### Cellular association of graphene nanoplatelets

The association of GNPs by macrophages was assessed via flow cytometry by evaluating shifts in cell side scatter (SSC) property (cell granularity/complexity), thereby qualitatively demonstrating the cellular uptake [20, 44]. In addition, to evaluate the role of scavenger receptor CD36 in facilitating the association of GNPs, sulfosuccinimidyl oleate (sodium salt) (SSO), a competitive ligand of the fatty acid translocase CD36, was used to pretreat the macrophages for 1 h at the concentration of 100 μM, prior to the GNPs exposures. SSO remained in the SFM during the GNPs exposures. Macrophages were grown at 90% confluency in 24-well plates and the macrophages with or without the pretreatment of SSO were then exposed to 0, 50, or 100 μg/mL of GNPs for 1 or 3 h in SFM. Besides SSO, a goat anti-mouse CD36/SR-B3 polyclonal antibody (R&D systems, Catalog number AF2519, Minneapolis, MN) and 2-(2-butoxyethyl)-1-cyclopentanone thiosemicarbazone (BLT2), an inhibitor of scavenger receptor-B1 (SR-B1), were also used to block the potential interactions mediated by CD36 and SR-B1 receptors. Macrophages were pretreated with the CD36 antibody at a concentration of 2.5 μg/10^6^ cells or 50 μM BLT2 for 1 h followed by a 2h-exposure to 100 μg/mL GNPs. The CD36 antibody remained in the SFM during the GNP exposure, while BLT2 was removed before GNP exposure. Macrophages exposed to 100 μg/mL of GNPs but without the pretreatment of SSO, CD36 antibody, or BLT2 were used as positive control. At the end of exposures, macrophages were collected and analyzed using flow cytometry (Accuri C6 Flow Cytometer, BD Biosciences, San Jose, CA) to determine the shift in SSC.

### Mitochondrial membrane potential

Often alterations in mitochondrial endpoints are early events in cellular toxicity. To evaluate alterations in mitochondrial function, macrophages cultured in the 24-well plates were pretreated with CD36 antibody at the concentration of 2.5 μg/10^6^ cells or 50 μM BLT2 for 1 h prior to the 2 h-exposure to 100 μg/mL GNPs. Untreated macrophages were considered as the negative control, macrophages without pretreatment but exposed to 100 μg/mL of GNPs or 100 μg/mL of ZnO NPs for 2 h were used as the exposure control or the positive control, respectively. At the end of the exposures, the culture medium was removed and the cells were washed with PBS three times followed by the incubation with 10 μM Rhodamine 123 (Rh123) (Thermo Fisher Scientific, Grand Island, NY) in PBS at 37°C for 15 min. Macrophages were pelleted and re-suspended in 0.5 mL PBS after three washes with PBS. The cell suspension samples were then loaded into the black 96-well plate and read at 480 nm (Excitation)/520 nm (Emission) using a plate reader. The BCA protein assay was used to determine the protein concentration of the cellular samples. The intracellular Rh123 levels were normalized by protein quantity and expressed as cellular fluorescent intensity/μg protein.

### Metabolomics sample preparation and extraction

Macrophages were grown to 90% confluency in 24-well plates and the macrophages were exposed to 0, 50, or 100 μg/mL GNPs for 1 h or 3 h (for 0 and 50 μg/mL GNPs) in SFM or to 100 μM of a CD36 ligand (SSO) for 1 h. Three biological replicates were performed. At the end of the exposures, cell culture medium was removed and macrophages were washed three times with ice cold PBS. Protein removal and sample extraction were performed by adding 500 μL acetonitrile to 200 μL of cell matrix. Solutions were sonicated for 10 min. Water (500 μL) was added and samples were shaken for 5 min, followed by centrifugation at 16,000 × *g* for 8 minutes. The supernatants were transferred to separate vials and evaporated to dryness in a vacuum concentrator. The dried polar fractions were reconstituted in 100 μL of diluent composed of 80% water and 20% acetonitrile, containing 0.1% formic acid.

### High performance liquid chromatography-mass spectrometry (HPLC-MS) and bioinformatics analyses

Separations were performed on an Agilent 1290 system (Palo Alto, CA), with a mobile phase flow rate of 0.45 mL/min. The metabolites were assayed using a Waters HSS T3 column (1.8 μm, 2.1 × 100 mm), where the mobile phases A and B were 0.1% formic acid in ddiH_2_O and acetonitrile, respectively. Initial conditions were 100:0 A:B, held for 1 minute, followed by a linear gradient to 70:30 at 16 min, then 5:95 at 21 min. Column re-equilibration was performed by returning to 100:0 A:B at 22 minutes and holding until 27 minutes. The mass analysis was obtained using an Agilent 6545 Q-TOF mass spectrometer with ESI capillary voltage +3.2 kV, nitrogen gas temperature 325°C, drying gas flow rate 8.0 L/min, nebulizer gas pressure 30 psig, fragmentor voltage 135 V, skimmer 45 V, and OCT RF 750 V. Mass data (from m/z 70–1000) were collected using Agilent MassHunter Acquisition software (v. B.06). Mass accuracy was improved by infusing Agilent Reference Mass Correction Solution (G1969-85001). MS/MS was performed in a Data-dependent Acquisition mode.

Peak deconvolution and integration was performed using Agilent ProFinder (v. B.06). Bioinformatics were performed using Agilent’s Mass Profile Professional (v. 13.1). Chromatographic peaks were aligned across all samples. Peak areas were normalized by converting to log2 and applying a 75% percentile shift. Significance analysis was performed by an unpaired t-test with Benjamini–Hochberg FDR correction. Metabolites with p < 0.01 and fold change > 2 were considered significant. Peak annotations were performed using the METLIN (www.metlin.scripps.edu) and HMDB (www.hmdb.ca) metabolite databases, with a mass error of less than 15 ppm. Compounds were identified based upon proposed structures determined by HPLC-MS data and searching the HMDB database. The metabolism pathways were constructed using PathVisio software and taking KEGG metabolism pathway maps for reference [45–47].

### Statistics

All data are presented as mean ± SEM and consist of 3–6 experiments. Comparisons of the differences among the control and GNP-exposed groups within the same time point were analyzed by one-way ANOVA with post hoc comparisons by Tukey test. All the statistical analyses were performed using GraphPad Prism 6 software (GraphPad, San Diego, CA). Statistical significance was determined when p value was found to be ≤ 0.05 between groups.

## Results and discussion

### Characterization of graphene nanoplatelets

GNPs can be characterized by many techniques including SEM, AFM and Raman spectroscopy due to their distinctive band structure and physical properties. The GNPs were dispersed in either water or SFM at 100 μg/mL. In order to investigate the morphology of the GNPs in different solutions, analysis by field-emission scanning electron microscopy (FESEM) was conducted. For the GNPs dispersed in water, the lateral size is in the range of several hundred nanometers to several micrometers with relatively flat surface as shown in Fig 2A. In addition, the stacked layers were observed indicating the multi-layered structure. Atomic force microscopy (AFM) was utilized to measure the thicknesses of the GNPs. It can be seen in Fig 2B, the lateral size and obvious stacking morphology are consistent with the results of FESEM. The line-scan profile corresponding to the blue line in Fig 2B demonstrates the thickness of 38.10 nm further confirming the two-dimensional (2D) forms of the GNPs. Moreover, Raman spectrum (S1 Fig) was performed to confirm the graphene structure. There are three Raman active peaks appearing at ~1350, ~1580 and ~2700 cm^-1^ which can be indexed as the D, G and 2D peaks, respectively. The peak positions are agreeing well with former reports verifying the existence of the graphene structure [48, 49]. Therefore, the morphology and structure of 2D GNPs dispersed in water were characterized and confirmed. Then, similar procedures were taken for the GNPs dispersed in SFM to compare the morphology differences in various solutions. Unlike the dispersibility observed in water, the GNPs dispersed in SFM were strongly aggregated which is probably due to the ions in solution absorbing onto the surface of the GNPs and reducing surface charge. GNPs were also found to be covered by crystals from nutrition salts in SFM solution by both SEM and AFM analysis (Fig 2C and 2D). The thickness measurement of GNPs was also influenced by the salt layer. The line-scan profile in Fig 2D showed the thickness above 800 nm with slant background. This assessment demonstrated that the GNPs used in our investigation have a 2D structure and are nanosized within water however in typical cell culture conditions due to the addition of salts the sizes are altered. This characterization was necessary in order to understand results from subsequent *in vitro* experiments.

### Cell viability, cellular association and mitochondrial membrane potential changes in macrophages following the exposure to graphene nanoplatelets

**Fig 3:**
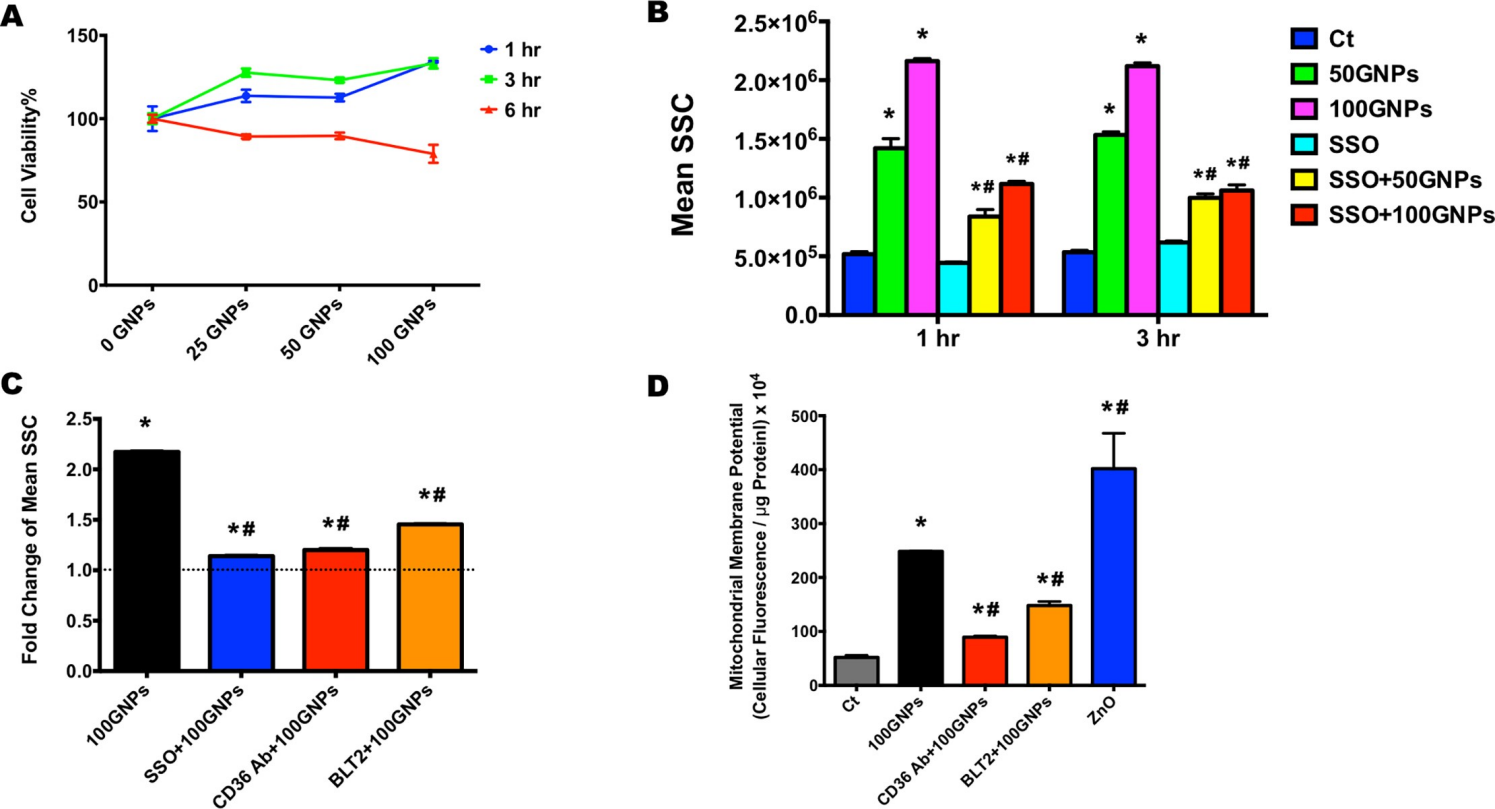
Cell viability, cellular uptake, and mitochondrial membrane potential changes following exposure to GNPs. (A). Cell viability percentages of macrophages following the exposure to 0, 25, 50, or 100 μg/mL GNPs for 1, 3, or 6 hr. Data represent mean ± SEM, n = 6/group. *: p < 0.05, as compared with the control group. (B). Cellular uptake of GNPs following the exposure to 50 or 100 μg/mL GNPs for 1 or 3 hr with or without the 1 hr-pretreatment of 100 μM SSO by side scatter (SSC) using flow cytometry. Data represent mean ± SEM, n = 6/group. *: p < 0.05, as compared with the control group; #: p < 0.05, as compared with the 50 μg/mL GNPs group. (C). SSC fold change of macrophages following the exposure to 100 μg/mL GNPs for 2 hr with or without the 1 hr-pretreatment of 100 μM SSO, CD36 antibody, or 100 μM BLT2. Data represent mean ± SEM, n = 6/group. *: p < 0.05, as compared with the control group; #: p < 0.05, as compared with the 100 μg/mL GNPs group. (D). Intracellular Rhodamine 123 (R123) accumulation in macrophages exposed to 0 or 100 μg/mL GNPs with or without the 1 hr-pretreatment of CD36 antibody or 100 μM BLT2. Macrophages exposed to 100 μg/mL ZnO NPs were used as positive control. Data represent mean ± SEM, n = 6/group. *: p < 0.05, as compared with the control group; #: p < 0.05, as compared with the 100 μg/mL GNPs group.

To determine whether GNP exposure induces cytotoxicity, macrophages were exposed to 0, 25, 50, or 100 μg/mL of GNPs for 1, 3, or 6 h in SFM and examined for differences in cell viability using the propidium iodide (PI) nucleic acid staining assay and flow cytometry analysis (Fig 3A). Macrophages exposed to 50 or100 μg/mL ZnO NPs were used as positive controls. No significant cytotoxicity was observed at the selected GNPs concentrations at any of the evaluated time points, while the 6 h-exposure to 50 or 100 μg/mL ZnO nanoparticles induced significant cell death (29.4 ± 6.5 and 52.4 ± 12.1%, respectively) as compared with the non-treated macrophages. Thus, exposure doses of 50 and 100 μg/mL GNPs and exposure time points of 1 and 3 h were selected for all subsequent experiments. Moreover, prior to assessing the cell viability of macrophages following GNPs exposure using PI staining assay, we used the traditional 3-(4,5-dimenthylthiazol-2-yl)-2,5-diphenyltetrazolium bromide (MTT) assay. Our MTT results showed significant reductions in the optical density after 1 h-treatment with 6.25 μg/mL GNPs (31.0 ± 0.6%) yet no visible cell death was observed under the light microscope. The MTT is an assay that indirectly reflects viable cell numbers by measuring the mitochondrial metabolic rate through the conversion of the tetrazolium salt MTT into formazan crystals [50]. A number of studies have shown that inhibitors, NPs, polypeptides and X-ray radiation impact the reduction rate of MTT leading to possible over or underestimation of cell viability [51–56]. Our MTT findings suggest that GNPs may interfere with the MTT reagent by changing the metabolic activity, thereby influencing the MTT absorbance measurement leading to underestimation of macrophage viability. Thus, when studying GNPs-induced cytotoxicity, the appropriate viability assay must be selected.

Scavenger receptors consist of a diverse array of membrane-bound receptors. Class B scavenger receptors comprise of SR-B1, CD36 (SR-B2) and SR-B3 which can recognize a variety of ligands including lipoproteins, cholesterol ester, oxidized phospholipid, apoptotic cells, bacterial and fungal pathogens [26, 57–60]. These scavenger receptors exhibit a wide range of functions including lipid transport, pathogen clearance, platelet activation/aggregation, apoptosis, angiogenesis, inflammation, etc [24–26, 61]. In addition, accumulative evidence has demonstrated the internalization of nanoparticles mediated by SR-B1 and CD36 in macrophages, such as iron oxide, silver, silica nanoparticles, and carbon nanotubes [20, 21, 23, 44, 62, 63]. To determine the cellular uptake of GNPs and evaluate the roles of scavenger receptors in facilitating the internalization of GNPs, macrophages were exposed to GNPs with or without pretreatments designed to inhibit scavenger receptors CD36 and SR-B1. When compared with the control macrophages without any treatment, macrophages exposed to 50 or 100 μg/mL GNPs appeared to readily internalize GNPs following the 1 h- and 3 h-exposures. No differences were determined between the 1 h and 3 h time points suggesting that macrophage uptake of GNPs occurs readily and reaches saturation quickly. Inhibition of CD36, by pretreatment with SSO, significantly reduced the uptake of GNPs by 41% (50 μg/mL GNPs, 1 h), 49% (100 μg/mL GNPs, 1 h), 35% (50 μg/mL GNPs, 3 h), and 50% (100 μg/mL GNPs, 3 h) (Fig 3B). Similar cellular uptake reductions were also observed when the macrophages were pretreated with CD36 antibody (45% reduction), or BLT2 (inhibition of SR-BI) (33% reduction) prior to the 2 h-exposure to 100 μg/mL GNPs, as compared to macrophages exposed to GNPs without pretreatment with receptor inhibitors (Fig 3C). These findings indicate GNPs interact with macrophages through the scavenger receptors, particularly through the CD36. Although CD36 mediates a variety of signaling pathways involved in many essential cellular processes, the underlying mechanisms remain poorly understood. Profiling the CD36-specific metabolome of macrophages in response to CD36 specific ligands and GNPs exposure may yield novel insights for potential ENMs-induced effects.

Following the interactions with scavenger receptors and internalization, GNPs may interfere with normal macrophage function. For clarifying the underlying mechanisms of GNP-induced toxicity, it is important to assess mitochondrial function. Energy metabolism is an essential process to maintain the normal cellular functions and is often modulated early following an exposure. To determine GNP-induced induced alterations in mitochondrial function, the mitochondrial membrane potential in macrophages was measured using the Rh123 staining. Rh123 is a sensitive tracer dye used to specifically evaluate the membrane potential of mitochondria [64]. Under physiological condition, the negative membrane potential across the mitochondrial inner membrane attracts and accumulates Rh123 [65, 66]. Loss of this negative potential, referred as depolarization, can reduce the retention of Rh123 within the mitochondria, while an overload of Rh123 suggests an increase in the mitochondrial membrane polarization, referred to as hyperpolarization [67, 68]. Our results clearly show that a 2 h-exposure to 100 μg/mL GNPs and ZnO significantly increased the Rh123 fluorescent intensity in macrophages by approximately 5 fold and 8 fold respectively (Fig 3D), as compared with that in the control group, suggesting increased mitochondrial membrane potential. Disruption of mitochondrial membrane potential is considered as an irreversible event resulting from oxidative stress leading to activation of apoptotic signaling [69–72]. Studies have proposed that oxidative stress and subsequent apoptosis induced upon graphene internalization are the main mechanisms of graphene-induced toxicity [4, 73, 74]. The overproduction of reactive oxygen species induced by graphene materials triggers the release of cytochrome c complex from the mitochondrial inner membrane which could lead to the alteration of mitochondrial transmembrane potential [4]. Further, the pretreatment of macrophages with a CD36 antibody or an inhibitor of SR-BI (BLT2), markedly mitigated the GNPs-induced Rh123 overload by 64% and 41% respectively (Fig 3D), implying that the activation of CD36 may interrupt the mitochondrial respiratory chain by triggering the upstream cell signaling pathways to modulate the mitochondrial potential. The blockage of CD36 may undermine the CD36 signal transduction pathways resulting in protection effect in preventing the mitochondrial injury, which requires further in-depth investigations. Our results demonstrate a disruption of mitochondrial membrane potential while no changes in cytotoxicity were observed. This is likely a result of the selected time points and/or concentrations utilized within our study.

### Dose-response metabolomics changes following exposure to graphene nanoplatelets

**Fig 4:**
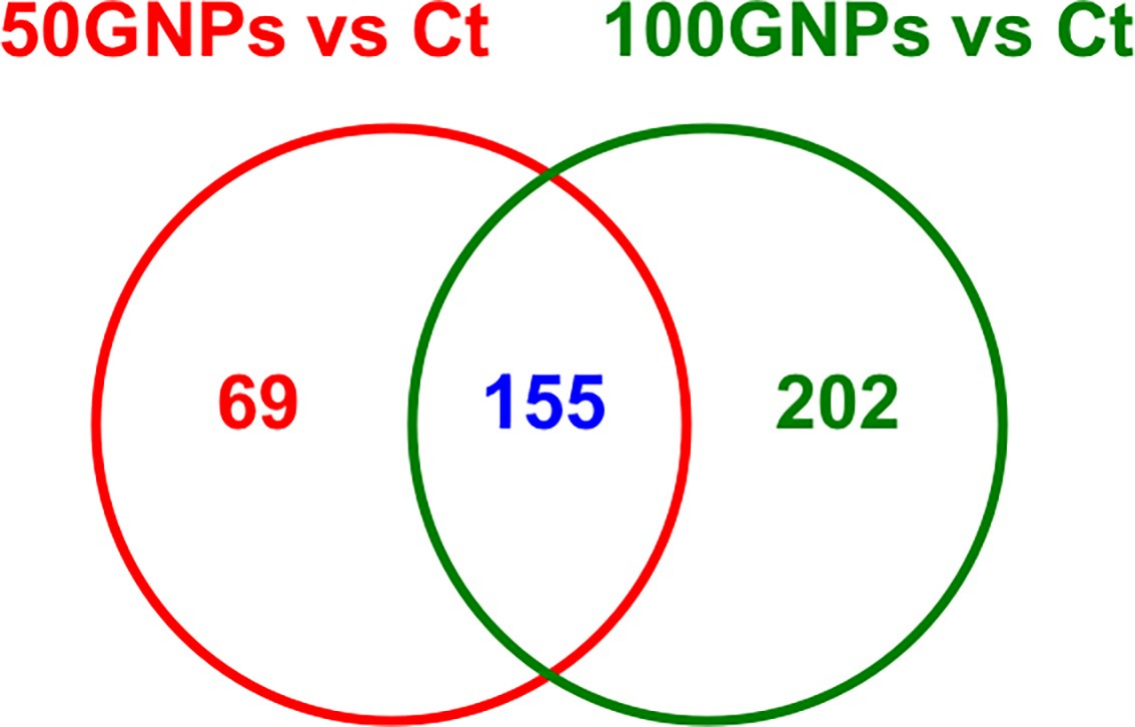
Venn diagram of dose-response metabolite changes between the comparisons of 50GNPs vs. control and 100 GNPs vs. control. Macrophages were exposed to 0 (control), 50, or 100 μg/mL GNPs for 1h followed by metabolomics analysis. Identified metabolites with fold changes greater than 2 and p value less than 0.01 are included within the diagram.

In order to profile dose-dependent alterations in the macrophage metabolome following exposure to GNPs, exposure concentrations of 50 and 100 μg/mL were tested and compared to non-treated control macrophages. Principle component analysis (PCA) is a well-established statistical tool used to emphasize variation, explore and visualize the grouping patterns in a dataset. The three dimensional PCA and Volcano plots in S2A Fig (50 μg/mL GNPs-exposed macrophages vs. non-treated control macrophages, 50GNPs vs. Ct), 2B (100 μg/mL GNPs-exposed macrophages vs. non-treated control macrophages, 100GNPs vs. Ct), and 2C (50 μg/mL GNPs-exposed macrophages vs. 100 μg/mL GNPs-exposed macrophages, 50GNPs vs. 100 GNPs) show clear separations between groups in terms of metabolite profiles following exposure to GNPs. When performing the comparisons of 50GNPs vs. Ct and 100GNPs vs. Ct, there were 224 (135 down-regulated and 89 up-regulated metabolites) and 357 (181 down-regulated and 176 up-regulated metabolites) metabolites showed significant changes (Fold Change > 2; p < 0.05) among a total of 3096 and 3315 metabolites detected, respectively (Table 1, S1 and S2 Tables). These results indicate that more metabolites are down regulated as compared with those are up-regulated, and as the exposure concentration of GNPs increases, more metabolites are found to be changed. Further, when processing the log fold change analysis, our Venn diagram in Fig 4 clearly displays that 155 metabolites were found to have similar alteration following the exposure to 50GNPs and 100GNPs as compared with the corresponding control, among the compounds detected by HPLC-MS using retention time and mass (Fig 4). Exposure to 50GNPs resulted in 69 metabolites uniquely altered compared to controls whereas 202 metabolites were unique to macrophages exposed to 100GNPs (Fig 4). This finding suggests dose-dependent metabolite changes exist following the exposure to GNPs.

**Table 1:** GNPs induces dose-response changes in metabolites.

Comparison	Total Metabolites	p<0.01; FC>2	Down-Regulated	Up-Regulated
50GNPs vs Ct	3096	224	135	89
100GNPs vs Ct	3315	357	181	176
50GNPs vs 100GNPs	5427	109	40	69

Note: Ct: control; 50GNPs: 50 μg/mL GNPs; 100GNPs: 100 μg/mL GNPs; FC: fold changes.

Metabolites found to be significantly modified (S1 and S2 Tables) were utilized to determine cellular metabolism pathways affected by GNPs exposure. A subset of these metabolites are included in Table 2. A number of metabolites were determined to be altered similarly between 50GNPs vs. Ct and 100GNPs vs. Ct. These metabolites were determined to have comparable fold changes in respect to controls. Further, these metabolites were identified to mainly associate with 5 metabolism pathways such as glutathione metabolism (i.e., γ-Glu-Cys, pyroglutamic acid, and NADPH), pantothenate and coenzyme A (CoA) biosynthesis (i.e., D-4’-Phosphopantothenate, pantetheine, pantothenic acid, and adenosine 3',5’-bisphosphate (PAP)), arachidonic acid metabolism (i.e., 5S-HETE-d8, 20-hydroxy-LTE4), purine metabolism (i.e., guanine and uric acid) and riboflavin metabolism (i.e., riboflavin and lumichrome) (Table 2). In addition, separate subsets of metabolites were determined to have unique alterations following the exposure to either 50GNPs or 100GNPs as compared to the corresponding controls (Table 2). Together these patterns suggest differential metabolic outcomes in macrophages exposed to GNPs based on concentration. Furthermore, levels of a number of glycerphospholipids, fatty acyls, and sterol lipids were distinguished in macrophages exposed to both concentrations of GNPs and most of these lipids were markedly down regulated (S1 Table). Dose-dependent effects were also observed in a variety of lipid compounds categorized as glycerophospholipids, glycerolipids, and sphingolipids (S1 Table). These findings imply that GNPs may be capable of influencing the lipid metabolism of macrophages, particularly the metabolism of glycerolipids, clycerophospholipids, and sphingolipids.

**Table 2:** Selected relevant metabolites to examine GNPs concentration-dependent responses.

Metabolite Category	Metabolite	50GNPs vs Ct		100GNPs vs Ct	
		p-Value	Log FC	p-Value	Log FC
Glutathione Metabolism	γ-Glu-Cys	8.03E-07	20.86	7.13E-08	20.21
	Pyroglutamic acid	2.63E-08	19.26	2.91E-08	20.38
	NADPH	6.31E-06	17.75	2.87E-07	17.99
	S-Nitroso-L-glutathione	1.46E-07	-17.79	1.13E-07	-17.34
Pyruvate metabolism	(R)-S-Lactoylglutathione	9.64E-09	-16.99	3.40E-09	-16.52
Pantothenate & CoA Biosynthesis	D-4'-Phosphopantothenate	4.22E-08	-18.71	2.38E-08	-18.27
	Pantetheine	9.13E-07	-17.55	8.63E-07	-17.10
	Pantothenic Acid	1.83E-07	16.86	3.04E-07	17.44
	Adenosine 3',5'-bisphosphate (PAP)	5.56E-08	17.66	5.92E-08	18.44
Arachidonic Acide Metabolism	5S-HETE-d8	5.03E-06	-18.32	5.31E-06	-17.87
	20-hydroxy-LTE4	7.57E-08	17.15	6.99E-08	17.69
Purine Metabolism	Guanine	2.22E-07	-17.30	1.77E-07	-16.86
	Uric acid	2.86E-04	-2.08	2.23E-04	-2.67
Riboflavin Metabolism	Riboflavin (Vitamin B2)	6.35E-05	2.50	6.01E-08	17.06
	Lumichrome	8.57E-08	17.17	4.63E-08	18.29
	Valyl-Gamma-glutamate	1.23E-08	-20.10	4.01E-09	-19.65
	Cholic acid glucuronide	1.12E-07	-18.69	8.40E-08	-18.24
Phenylpropanoid biosynthesis	N1,N5,N10-Triferuloyl spermidine	3.17E-07	-17.88	2.73E-07	-17.44
Argine and proline metabolism	N4-Acetylaminobutanal	7.49E-06	-17.70	7.83E-06	-17.26
Skimate/acetate-malonate pathway derived compounds	Isobatatasin I	1.43E-08	-16.99	3.08E-09	-16.55
	CPA(18:0/0:0)	3.21E-06	17.07	4.47E-07	17.23
	Prolyl-Lysine	9.14E-08	17.27	1.51E-07	18.02
	S-2-(Indol-3-yl)acetyl-CoA	3.41E-08	-18.60	No Change	
	Methionyl-Cysteine	1.22E-05	-18.04	No Change	
Tryptophan Metabolism	Xanthurenic acid	1.51E-04	-2.40	No Change	
Pyrimidine Metabolism	Uridine	4.40E-05	-1.50	No Change	
Ubiquinone and other terpenoid-quinone biosynthesis; Vitamin digestion and absorption	Phylloquinone	8.89E-07	18.30	No Change	
	Anandamide (22:6, n-3)	5.82E-07	20.40	No Change	
Folate biosynthesis; One carbon pool by folate; vitamin digestion and absorption	Folic acid	2.10E-08	20.41	No Change	
	PE-Cer(d14:2(4E,6E)/20:0(2OH))	No Change		9.91E-10	-19.23
Sphingolipid Metabolism	C16 Sphinganine	No Change		8.29E-10	-17.49
	Histidinyl-Leucine	No Change		2.07E-07	-17.47
Purine Metabolism	Hypoxanthine	No Change		6.30E-04	1.72
	Norepinephrine (noradrenaline)	No Change		9.47E-04	2.26
	Tyrosyl-Aspartate	No Change		5.43E-08	16.84
	5'-guanylate diphosphate	No Change		4.69E-08	16.96
Cystein and Methionine Metabolism	D-Cysteine	No Change		7.94E-08	17.17
	Gamma Glutamylglutamic acid	No Change		4.74E-07	17.41
	Tyrosyl-Gamma-glutamate	No Change		9.87E-07	17.54
Metabolism of xenobiotics by cytochrome P450	S-(2-Hydroxyethyl)glutathione	No Change		9.04E-07	17.70
Limonene and pinene degradataion; Degradation of aromatic compounds	3-Isopropenylpimelyl-CoA	No Change		4.11E-08	18.14
Naphthalene degradation; microbial metabolism in diverse environments; Degradation of aromatic compounds	2-Naphthoyl-CoA	No Change		3.86E-08	18.16

Note: Ct: control; 50GNPs: 50 μg/mL GNPs; 100GNPs: 100 μg/mL GNPs.

**Fig 5:**
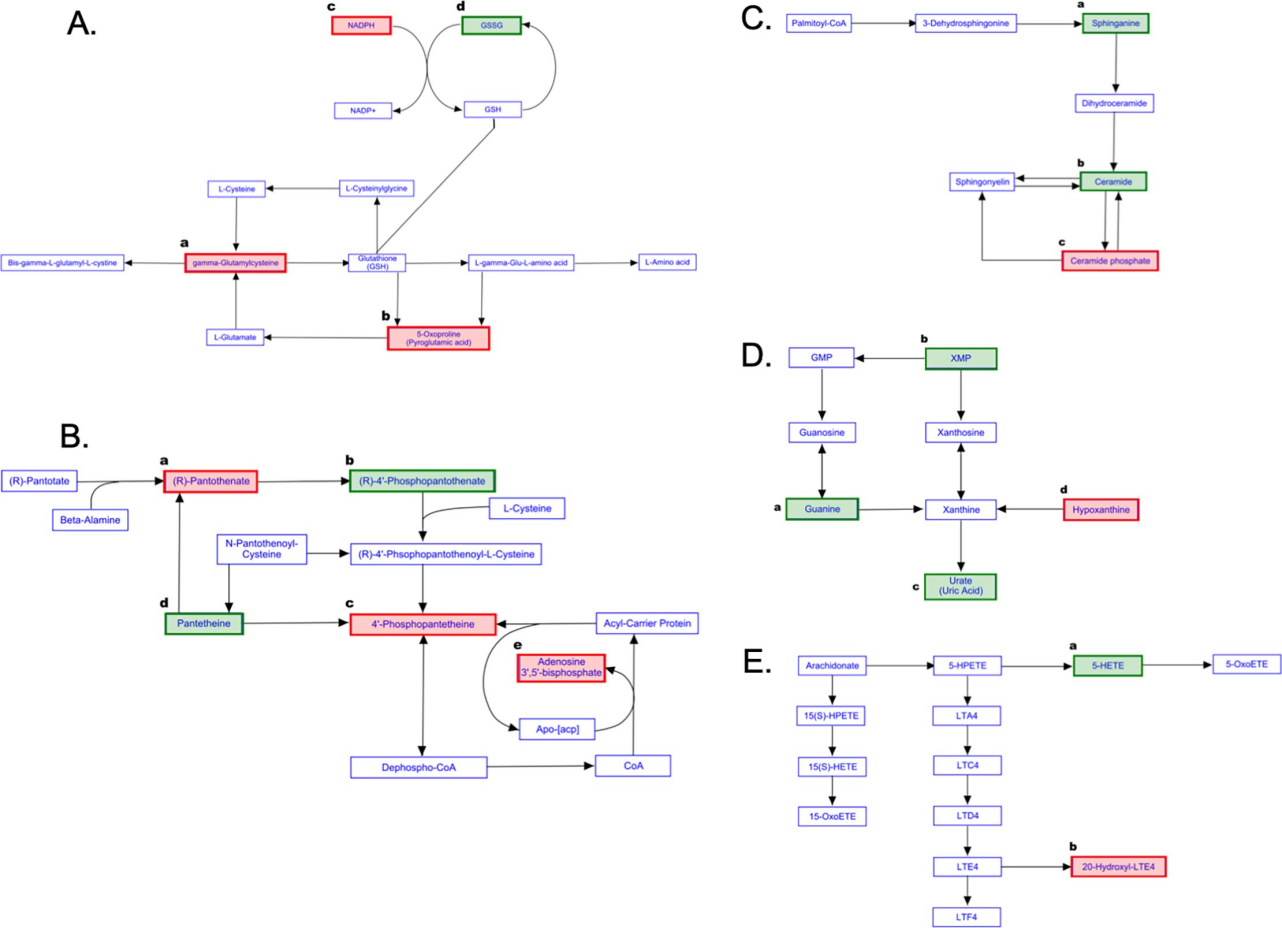
Metabolism pathways influenced following GNPs exposure. (A) Glutathione metabolism pathway: (a) gamma-glutamylcysteine (γ-Glu-Cys), (b) 5-Oxoproline, and (c) NADPH highlighted with red rectangular boxes were up-regulated following exposure to 50 or 100 μg/mL GNPs for 1h. In addition, γ-Glu-Cys was also increased following the exposure to 50 μg/mL GNPs for 3h. (d) GSSG (oxidized glutathione) highlighted with green rectangular box was significantly down-regulated following 1h-treatment with the CD36 ligand (100 μM SSO). (B) Pantothenate and CoA biosynthesis pathway: (a) (R)-Pantothenate (also named pantothenic acid) highlighted with the red rectangular box was the statistically up-regulated metabolite in both 50 and 100 μg/mL GNP-exposed macrophages for both 1h and 3h time points, as well as the 100 μM SSO-treated macrophages. (b) (R)-4’-Phosphopantothenate (also named D-4’-Phosphopantothenate) highlighted with the green rectangular box was the statistically reduced metabolite in all treatment groups. (c) 4’-Phosphopantetheine (also named D-Pantetheine 4'-phosphate) highlighted with the red rectangular box was the markedly up-regulated metabolite following the 3h-exposure to 50 μg/mL GNPs. (d) Pantetheine highlighted with the green rectangular box was the significantly down-regulated metabolite in all treatment groups except the group with 3h exposure to 50 μg/mL GNPs. (e) Adenosine 3’,5’-bisphosphate (PAP) highlighted with the red rectangular box was the significantly increased metabolite in both 50 and 100 μg/mL GNPs-exposed macrophages for both 1h and 3h time points. (C) Sphingolipid metabolism pathway: (a) Sphinganine highlighted with the green rectangular box was the significantly down-regulated metabolite in macrophages exposed to 100 μM SSO. (b) Ceramide (also named C16 Sphinganine) highlighted with the green rectangular box was the statistically reduced metabolite in macrophages exposed to 100 μg/mL GNPs. (c) Ceramide phosphate (also named CerP (d18:0/16:0)) highlighted with red rectangular box was the markedly up-regulated metabolites in 100 μg/mL GNPs-exposed macrophages. (D) Purine metabolism pathway: (a) Guanine highlighted in the green rectangular box was the significantly down-regulated metabolite in macrophages exposed to 50 or 100 μg/mL GNPs for 1h, as well as 100 μM SSO. (b) Xanthosine monophosphate (XMP) highlighted with the green rectangular box was the metabolite found to be down-regulated in macrophages pretreated with 100 μM SSO. (c) Urate (also named Uric acid) highlighted in the green rectangular box was the statistically reduced metabolite in macrophages exposed to 50 or 100 μg/mL GNPs for 1h. (d) Hypoxanthine highlighted with the red rectangular box was the markedly up-regulated metabolite in macrophages exposed 100 μg/mL GNPs for 1h and 50 μg/mL GNPs for 3h. (E) Arachidonic acid metabolism pathway: (a) 5-HETE (also named 5S-HETE-d8) highlighted with the green rectangular box was the statistically down-regulated metabolite in both 50 and 100 μg/mL GNP-exposed macrophages for both 1h and 3h time points. (b) 20-Hydroxyl-LTE4 highlighted with the red rectangular box was the statistically up-regulated metabolite in both 50 and 100 μg/mL GNP-exposed macrophages for both 1h and 3h time points.

Oxidative stress is one of the primarily proposed mechanisms of graphene toxicity, which can damage proteins, DNA, and lipids leading to progression of a number of diseases [9, 10]. The three identified biomarkers of the glutathione (GSH) metabolism pathway (Fig 5A) consisting of γ-Glu-Cys, pyroglutamic acid, and NADPH were up regulated following exposure to GNPs at both concentrations (Table 2) [75]. These findings suggest that the glutathione pathway may be activated in response to an increased demand for cellular glutathione to cope with an oxidative insult induced by GNPs. A stable GSH homeostasis plays essential roles in antioxidant defense, various metabolism, and regulation of diverse cellular events such as gene expression, DNA and protein synthesis, cell proliferation and apoptosis, signal transduction, cytokine production and immune response, protein glutathionylation, and mitochondrial function and integrity [76–81]. A few studies have been performed to evaluate the oxidative stress induced by graphene family nanomaterials, particularly graphene oxide, which have been found to be internalized in the cytosol and result in the increase in intracellular ROS levels in HepG2 cells, mouse embryo fibroblasts, and human lung fibroblast cells [42, 82–86]. To date, limited research is conducted to specifically evaluate the cellular toxicity induced by GNPs not to mention to investigate the underlying mechanisms. As GSH metabolism pathway is influenced by GNP exposure, further in-depth studies are required to validate the impact of GNPs on this pathway such as alterations in GSH activity, oxidized GSH (GSSG) to GSH ratio, levels of other metabolites related to oxidative balance, and interventional approaches.

Pantothenate and CoA biosynthesis is another major metabolism pathway that was disrupted following exposure to GNPs. Four identified metabolites of D-4’-phosphopantothenate (also named (R)-4’-phosphopantothenate), pantetheine, pantothenic acid (also named (R)-pantothenate or Vitamin B5), and adenosine 3’, 5’-bisphosphate (PAP, also named 3’-phosphoadenylate) are essential components of the pantothenate and CoA biosynthesis pathway [87]. These four identified metabolites along with ten other metabolites of CoA, Dephospho-CoA, Apo-[acp], acyl-carrier protein, 4’-phosphopantetheine (also named D-panthetheine 4’-phosphate), (R)-4’-phsphospantothenoyl-L-cysteine, N-pantothenoylcysteine, L-cysteine, (R)-Pantotate, and beta-alamine, were used to construct a partial and potential network of the pantothenate and CoA biosynthesis pathway (Fig 5B). According to our metabolomics data, macrophages exposed to both 50 and 100 μg/mL GNPs showed marked down-regulation of D-4’-phosphopantothenate and pantetheine, while the levels of pantothenic acid and adenosine 3’, 5’-bisphosphate were dramatically increased (Table 2). Pantothenic acid (Vitamin B5) is an essential nutrient for all living organism and the pivotal substrate for the synthesis of the ubiquitous CoA and the synthesis and metabolism of proteins, carbohydrates, and fats [88–90]. CoA, an essential cofactor and a universal acyl carrier, plays critical functions in a variety of metabolic reactions, such as the synthesis of phospholipids, synthesis and degradation of fatty acids, energy production through the tricarboxylic acid cycle, and regulation of lipid metabolism [88, 91–93]. The imbalance of key metabolites including D-4’-phosphopantothenate, pantetheine, pantothenic acid, and adenosine 3’, 5’-bisphosphate during the CoA biosynthesis induced by GNPs exposure implies that normal CoA biosynthesis was disrupted leading to abnormal production of CoA, which may subsequently interrupt the synthesis and degradation of fatty acids, energy production and lipid metabolism. Further in-depth studies are required to validate these biomarkers, which may also serve as the underlying mechanisms of GNPs-induced immunotoxicity.

Several other identified metabolites were significantly altered following GNPs exposure and found to participate in the pathways of sphinolipid metabolism (i.e, C16 Sphinganine (also named ceramide) and CerP (d18:0/16:0) (also named ceramide-1-phosphate or ceramide phosphate) (Fig 5C; [94]), and arachidonic acid metabolism (i.e., 5S-HETE-d8 (also named 5-HETE) and 20-Hydroxy-LTE4 (Fig 5E; [95]) (Table 2). Among these metabolites, no changes were observed in C16 Sphinganine and CerP (d18:0/16:0) in macrophages exposed to the lower dose of GNPs, while C16 Sphinganine was significantly down-regulated and CerP (d18:0/16:0) was markedly up-regulated in macrophages exposed to the higher concentration of GNPs (Table 2), suggesting concentration-dependent differential metabolite profiles induced by GNPs. Beyond serving as a major supporting structural lipid in the lipid bilayer of eukaryotic cells, sphingolipid also participates in modulating the cell fate and immune responses [96]. Ceramide and ceramide-1-phophate are two of the main bioactive sphingolipids involving in a number of cellular process including cell proliferation, migration, differentiation, apoptosis, cell cycle arrest, necrosis, autophagy, mitophagy, cytoskeleton rearrangement, insulin resistance, cellular metabolism, and regulation of inflammation [97–99]. Dis-regulation of these two bioactive sphingolipids may due to the abnormal metabolism of sphingolipids, which could lead to subsequent cellular dysfunctions described above. GNPs are well known to have sharp edges internalization of these particles may induce physical damages to the cellular lipid bilayer membrane leading to abnormal breakdown of membrane lipids. In addition, our data showed that GNPs induced hyperpolarization of the mitochondrial membrane (Fig 2D). Accumulating evidence show that ceramide acts within the mitochondria and cellular stressors can influence the generation of ceramide that could disrupt the normal mitochondrial function leading to apoptosis [100–103]. In addition to sphingolipid metabolism, arachidonic acid metabolism also belongs to the category of lipid metabolism. Two biomarkers (5-HETE and 20-Hydroxy-LTE4) were identified in the current study associated with the latter metabolism pathway suggesting that GNPs exposure interferes with the lipid metabolism. Therefore, further studies are needed to explore the impact of GNPs on the cellular lipid metabolism.

Guanine, Uric acid, and Hypoxanthine identified by our metabolomics analysis are associated with the purine metabolism [104]. The first two metabolites were markedly down regulated by both concentrations of GNPs, while hypoxanthine was significantly induced only by the higher concentration of GNPs (Table 2, Fig 5D). Purines are the basic structural components for DNA and RNA involving in the regulation of cellular metabolism, energy conservation and transport, metabolism of lipid and carbohydrate, signal transduction and translation [105–107]. Guanine, a universal nucleo purine, and Uric acid, the most important oxidized purine and the major end-product of purine metabolism, are the key components in the breakdown process of purine metabolism, and reductions of these two metabolites might indicate a suppression of the breakdown of purine following GNP exposure [108, 109]. Hypoxanthine, on the other hand, is a hydroxyl purine presents in tissues and bio-fluids of animals and serves as an essential metabolite in the salvage process of purine metabolism [108, 109]. The accumulation of hypoxanthine caused by the high concentration of GNPs appears to parallel with the reduction of Uric acid suggesting a comprised reutilization of hypoxanthine that ultimately lead to a substantial reduction in the breakdown of purines. To validate effects of GNPs on purine metabolism, future studies are necessary to evaluate activities of related enzymes and levels of associated metabolites.

### Time-course metabolomics changes following the exposure to graphene nanoplatelets

**Fig 6:**
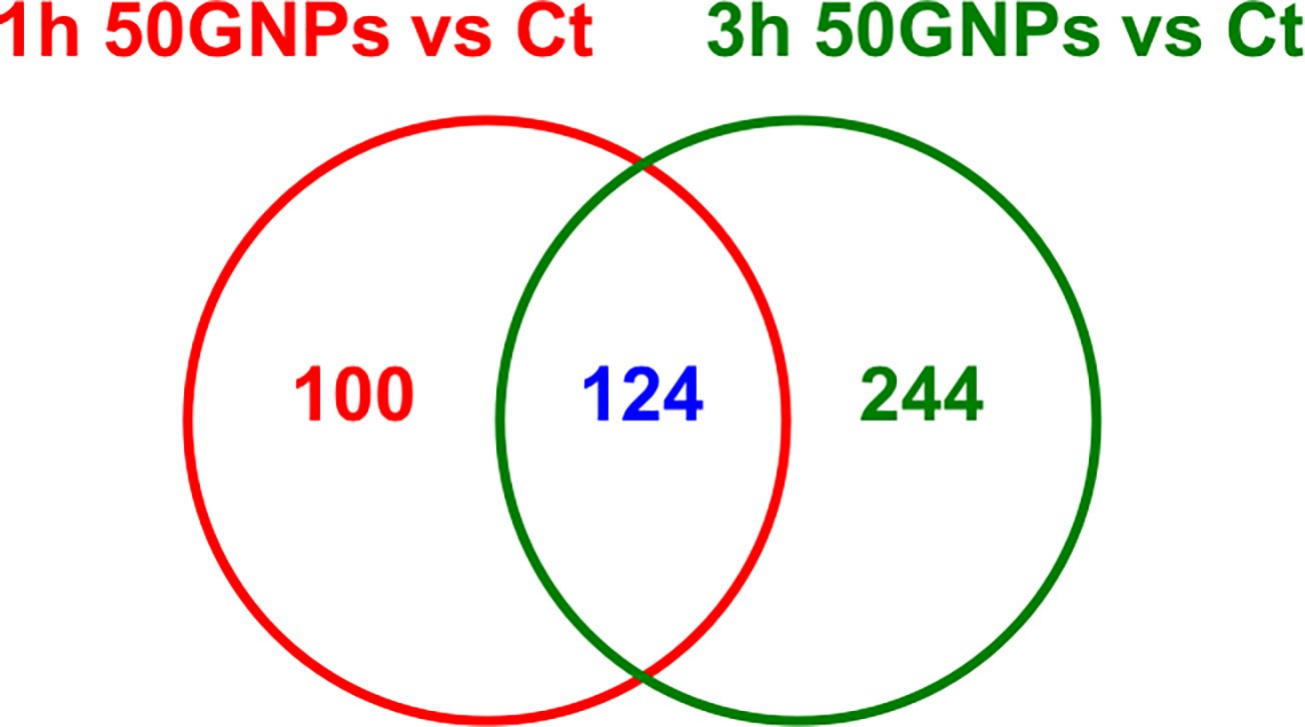
Venn diagram of time-course metabolite changes between the comparisons of 1h 50GNPs vs. control and 3h 50GNPs vs. control. Macrophages were exposed to 0 (control) or 50 μg/mL GNPs for 1 or 3h followed by metabolomics analysis. Identified metabolites with fold changes greater than 2 and p value less than 0.01 were are included within the diagram.

In order to examine time-dependent changes in metabolite profiles, macrophages were exposed to 50 μg/mL GNPs for 1 h or 3 h. A clear separation in metabolite profiles due to different exposure time points (1h vs. 3h) were observed in the three dimensional PCA while Volcano plots demonstrated altered cellular metabolites (S3 Fig). As the exposure time increased, the number of altered metabolites was also increased, a total of 368 metabolites were significantly changed (Fold Change > 2; p < 0.05) following a 3h-exposure to 50 μg/mL GNPs as compared with a total of 224 metabolites that were significantly changed at 1h (Table 3). During the 1h-exposure, 135 metabolites were determined to be down-regulated while 89 metabolites were up regulated. Assessment at 3 h however demonstrated 189 metabolites were up regulated and 179 metabolites were down regulated (Table 3). These data suggest a time-dependence associated with GNP-induced metabolism changes in macrophages. The Venn diagram in Fig 6 reveals that 124 metabolites among all significantly altered metabolites were shared by macrophages exposed to 50 μg/mL of GNPs at 1h and 3h. Further there were 100 metabolites unique to the 1h exposure time point while 244 were unique to the 3h time point.

**Table 3:** GNPs induces time-course changes in metabolites.

Comparison	Total Metabolites	p<0.01; FC>2	Down-Regulated	Up-Regulated
1h 50GNPs vs Ct	3096	224	135	89
3h 50GNPs vs Ct	3565	368	179	189
3h 50GNPs vs 1h 50GNPs	3095	225	122	103

Note: Ct: control; 50GNPs: 50 μg/mL GNPs; FC: fold change.

Among the detected and significantly altered compounds identified from metabolomic profiling, a subset was selected and is presented within Table 4 to examine time-dependent responses (S1 and S3 Tables). Many metabolites were altered at both 1 h and 3 h whereas others were found to be altered only at one of the selected time points (Table 4). Taken together, the shorter time point of GNP exposure appeared to influence fewer metabolites changes compared to the longer time point. Proportionally of these altered metabolites more were found to be up-regulated at the 3 h exposure as compared to the 1 h treatment. These findings suggest that GNPs tend to result in differential metabolomics profiles in a time-dependent manner. Although the major affected metabolism pathways were similar to the dose-response metabolomics profiles including glutathione metabolism, pantothenate and CoA biosynthesis, purine metabolism, and arachidonic acid metabolism (Table 4 and Fig 5A, 5B, 5D and 5E), time-course associated differential alterations were observed in metabolites related to these pathways similar to the dose-response findings. These findings suggest that the exposure time span could result in differential macrophage responses following GNPs exposure leading to altered metabolomics profiles. Therefore, multiple exposure time points should be included in the evaluation of GNPs-induced toxicity.

**Table 4:** Selected relevant metabolites to demonstrate time-course responses to GNP exposure.

Metabolite Category	Metabolite	50GNPs vs Ct (1 hr)		50GNPs vs Ct (3 hr)	
		p-Value	Log FC	p-Value	Log FC
Glutathione Metabolism	γ-Glu-Cys	8.03E-07	20.86	1.67E-08	22.78
	(R)-S-Lactoylglutathione	9.64E-09	-16.99	2.34E-06	18.23
Pantothenate & CoA Biosynthesis	D-4'-Phosphopantothenate	4.22E-08	-18.71	2.49E-08	-18.14
	Pantothenic Acid	1.83E-07	16.86	3.48E-07	18.14
	Adenosine 3',5'-bisphosphate (PAP)	5.56E-08	17.66	9.84E-08	18.52
Arachidonic Acide Metabolism	5S-HETE-d8	5.03E-06	-18.32	5.78E-06	-17.75
	20-hydroxy-LTE4	7.57E-08	17.15	3.39E-08	17.71
	Valyl-Gamma-glutamate	1.23E-08	-20.10	4.80E-09	-19.53
	S-2-(Indol-3-yl)acetyl-CoA	3.41E-08	-18.60	2.03E-08	-18.03
Argine and proline metabolism	N4-Acetylaminobutanal	7.49E-06	-17.70	7.94E-06	-17.13
	Retinyl beta-glucuronide	6.07E-09	-17.20	9.35E-10	-16.63
Skimate/acetate-malonate pathway derived compounds	Isobatatasin I	1.43E-08	-16.99	4.87E-09	-16.42
Riboflavin Metabolism	Riboflavin (Vitamin B2)	6.35E-05	2.50	7.53E-08	-16.88
Folate biosynthesis; One carbon pool by folate; vitamin digestion and absorption	Folic acid	2.10E-08	20.41	4.73E-08	20.74
	Methionyl-Cysteine	1.22E-05	-18.04	No Change	
	S-Nitroso-L-glutathione	1.46E-07	-17.79	No Change	
Pantothenate & CoA Biosynthesis	Pantetheine	9.13E-07	-17.55	No Change	
	Lysyl-Isoleucine	3.54E-08	-17.41	No Change	
	3-Methylene-indolenine	1.08E-07	-17.16	No Change	
Purine Metabolism	Guanine	2.22E-07	-17.30	No Change	
	Uric acid	2.86E-04	-2.08	No Change	
Tryptophan metabolism	Xanthurenic acid	1.51E-04	-2.40	No Change	
Pyrimidine Metabolism	Uridine	4.40E-05	-1.50	No Change	
	Tryptophyl-Glycine	4.07E-04	-1.25	No Change	
	20-oxo-heneicosanoic acid	5.13E-08	16.95	No Change	
	CPA(18:0/0:0)	3.21E-06	17.07	No Change	
	Prolyl-Lysine	9.14E-08	17.27	No Change	
Glutathione Metabolism	NADPH	6.31E-06	17.75	No Change	
	Pyroglutamic acid	2.63E-08	19.26	No Change	
Arginine biosynthesis	L-Arginine	No Change		9.98E-09	-18.88
	Leucyl-Glutamate	No Change		2.09E-08	-17.45
	Isoleucyl-Glutamate	No Change		1.02E-08	-16.92
Neuroactive ligand-receptor interaction	Endomorphin-2	No Change		1.30E-07	-16.65
	Norepinephrine (noradrenaline)	No Change		1.66E-04	3.23
Pantothenate & CoA biosynthesis	D-Pantetheine 4'-phosphate	No Change		8.84E-08	16.74
	6-Hydroxy-5-methoxyindole glucuronide	No Change		1.18E-07	16.81
	Tyrosyl-Asparagine	No Change		1.94E-07	16.89
	L-gamma-glutamyl-L-isoleucine	No Change		9.80E-08	17.10
Metabolism of xenobiotics by cytochrome P450	2,2-Dichloro-1,1-ethanediol	No Change		3.30E-06	17.31
Vitamin B6 metabolism Vitamin digestion and absorption	Pyridoxal (Vitamin B6)	No Change		5.22E-07	17.60
Cystein and Methionine Metabolism	D-Cysteine	No Change		1.71E-07	17.83
	Norepinephrine sulfate	No Change		1.26E-07	17.86
	Isoleucyl-Arginine	No Change		1.46E-07	17.87
Riboflavin Metabolism	Lumichrome	No Change		9.57E-08	17.92
	Tyrosyl-Gamma-glutamate	No Change		3.64E-07	17.93
Riboflavin Metabolism	2,5-Diamino-6-hydroxy-4-(5'-phosphoribosylamino)-pyrimidine	No Change		6.05E-07	17.96
Naphthalene degradation Degradation of aromatic compounds	2-Naphthoyl-CoA	No Change		1.22E-07	18.08
Antibiotic	Lymecycline	No Change		6.78E-08	18.13
	Erythronic acid	No Change		1.11E-05	18.24
Metabolism of xenobiotics by cytochrome P450	S-(2-Hydroxyethyl)glutathione	No Change		5.78E-08	18.43
Purine Metabolism	Hypoxanthine	No Change		9.47E-07	18.45
Limonene and pinene degradataion Degradation of aromatic compounds	3-Isopropenylpimelyl-CoA	No Change		4.07E-08	18.73

Note: Ct: control; 50GNPs: 50 μg/mL GNPs; FC: fold change.

Specifically, two metabolites (γ-Glu-Cys and (R)-S-Lactoylglutathione) of the glutathione metabolism were statistically increased in both 1h and 3h time points following the exposure to 50 μg/mL GNPs as compared with the corresponding control group (Table 4 and Fig 5A). While as the essential metabolites of glutathione metabolism, NADPH and Pyroglutamic acid were detected significantly raised only in 1h-GNPs-exposed macrophages (Table 4 and Fig 5A). This activated glutathione metabolism reflects the acute reaction of macrophages in response to the oxidative stress induced by GNPs, yet, as exposure time increases, this antioxidant defense system appears to be compromised which could result in the overload of ROS leading to subsequent apoptosis. Our metabolomics data showed that three of the five metabolites including D-4’-Phosphopantothenate (down-regulated), Pantothenic acid (up-regulated), and Adenosine 3’,5’-bisphosphate (up-regulated) that participate in Pantothenate and CoA biosynthesis pathway were markedly affected by GNPs in both 1 and 3h exposures (Table 4 and Fig 5B). Dramatic down-regulation in Pantethein was detected in 1h-GNPs-exposed macrophages while marked up-regulation in D-Pantetheine 4’-phosphate was observed in macrophages exposed to GNPs for 3h (Table 4). Exposure time-associated disruptions on these five key components of Pantothenate and CoA biosynthesis could interrupt the generation of CoA which may subsequently disturb the normal synthesis and degradation of phospholipids and fatty acids, energy production and the regulation of lipid metabolism [88, 91–93], however, the exact mechanism for these impacts is not yet known. Further, levels of guanine and uric acid were statistically reduced in macrophages exposed to 50 μg/mL GNPs for 1h but no changes were observed in the 3h-exposure (Table 4 and Fig 5D). Yet the hypoxanthine level was markedly increased when the GNPs exposure extended to 3h (Table 4 and Fig 5D). These exposure time-associated differential alterations in purine metabolism pathway might imply that GNPs exposure-compromised breakdown process appears earlier than the GNPs exposure-provoked salvage process during the purine metabolism. Similar to the dose-response changes in Table 2, the metabolites of 5S-HETE-d8 and 20-hydroxy-LTE4 showed the same trends of down-regulation and up-regulation, respectively, after both 1h- and 3h-exposures (Table 4), which could attribute to a disruption of lipid metabolism. In addition to the identified metabolites shown in Table 4, a large number of glycerophsopholipids, glycerolipids, fatty acyls, sphingolipids, and sterol lipids were detected in our metabolomics analysis and these lipids either were shared by both time point exposures or unique to either exposure time point (S1 and S3 Tables), which indicates that GNPs have significant influence on macrophage lipid metabolism deserving further investigation to explore the exact mechanism.

### CD36 ligand-associated changes in macrophage metabolism

**Fig 7:**
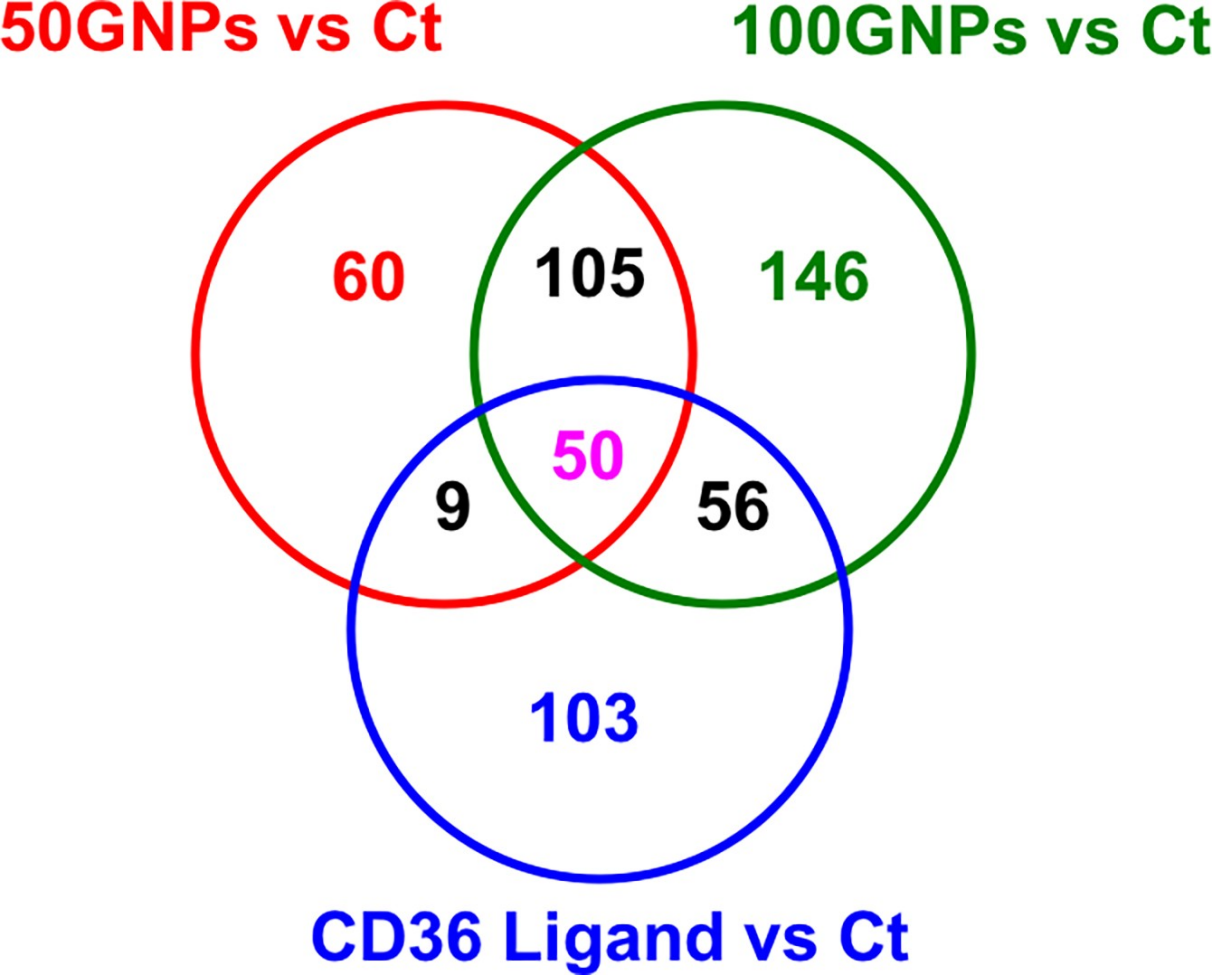
Venn diagram of CD36 ligand-associated metabolite changes among the comparisons of 50GNPs vs. control, 100 GNPs vs. control, and CD36 ligand vs. control. Macrophages were exposed to 0 (control), 50, or 100 μg/mL GNPs for 1h or to 100 μM of the CD36 ligand, SSO, followed by metabolomics analysis. Identified metabolites with fold changes greater than 2 and p value less than 0.01 are included within the diagram.

To further explore the role of CD36 in mediating macrophage interactions and toxicity following GNP exposure, the metabolomes of macrophages exposed to a CD36-specific ligand (SSO) were also evaluated and compared with metabolites altered following GNP exposures. Again, our PCA and Volcano plots reveal clear separation of metabolomic profile patterns due to the SSO exposure (S4 Fig). Comparisons of the number of metabolites detected following exposure can be found in Table 5, including total number detected, significantly altered, and directionality (Fold Change > 2; p < 0.05). Fewer metabolites were found to be significantly altered when the macrophages received the treatment with the CD36 ligand compared to those exposed to GNPs (Table 5). Moreover, treatment with the CD36 ligand also appeared to result in more down-regulated metabolites (CD36 Ligand vs. 50GNPs: 122 metabolites; CD36 Ligand vs. 100GNPs: 118 metabolites) than up-regulated metabolites (CD36 Ligand vs. 50GNPs: 86 metabolites; CD36 Ligand vs. 100GNPs: 39 metabolites) compared to GNP exposures (Table 5). These findings indicate that the activation of CD36 by the SSO triggers some CD36-associated signaling pathways that overlap with GNP exposure and some that do not. The Venn diagram in Fig 7 was constructed to correspond to the relationships shown in Table 5 demonstrating that: (1) 50 metabolites were altered similarly between the exposure of GNPs and activation of CD36 via SSO suggesting CD36 dependence of these responses, (2) as the concentration of GNPs increased so does the numbers of shared metabolites between GNP and SSO exposed macrophages suggesting that CD36 is more engaged by GNPs at the higher concentration; and (3) there are a number of metabolites that were uniquely modified only by GNP and not SSO, suggesting CD36 independent responses.

**Table 5:** GNPs and/or CD36 ligand induce changes in metabolites.

Comparison	Total	p<0.01; FC>2	Down-Regulated	Up-Regulated
CD36 Ligand vs Ct	3081	218	121	97
50GNPs vs Ct	3096	224	135	89
100GNPs vs Ct	3315	357	181	176
CD36 Ligand vs 50GNPs	5368	208	122	86
CD36 Ligand vs 100GNPs	2695	157	118	39

Note: CD36 Ligand: CD36 ligand SSO-pretreated macrophages; Ct: control macrophages; 50GNPs: 50 μg/mL GNPs-exposed macrophages; 100GNPs: 100 μg/mL GNPs-exposed macrophages; FC: fold change.

Significantly altered metabolites following exposure to GNPs or the CD36 ligand were examined to identify similar and dissimilar cellular metabolite biomarkers (S4–S6 Tables). A subset of this comparison is provided in Table 6 and suggests metabolites that may be CD36-dependent and -independent. Many of these metabolites were altered following exposure to both concentrations of GNPs as well as the CD36 ligand whereas others were only found to be shared when the GNPs were at the highest concentration. Overall, these affected metabolites participated in the metabolism pathways displayed in Fig 5A to 5E. Exposure to either dose of GNPs and SSO treatment significantly reduced the levels of D-4’-phosphopantothenate and pantetheine two main components in pantothenate and CoA biosynthesis pathway (Table 6 and Fig 5B) and the level of guanine a key metabolite in purine metabolism pathway (Table 6 and Fig 5D). Similar to the findings shown in Table 2, glutathione metabolism, pantothenate and CoA biosynthesis, arachidonic acid metabolism, and purine metabolism were the major metabolism pathways influenced by GNPs exposure (Table 6). Interestingly, the treatment of CD36 ligand SSO specifically decreased the levels of oxidized glutathione (also named glutathione disulfide (GSSG)), xanthosine monophosphate (XMP), and sphinganine involved in glutathione metabolism (Fig 5A), purine metabolism (Fig 5D) and sphingolipid metabolism (Fig 5C), respectively, these reductions were not detected in macrophages exposed to GNPs at different doses or different exposure time points (Tables 2, 4 and 6). These CD36 ligand-specific findings indicate that activation of CD36 may trigger or disable CD36-associated signaling transduction leading to differential responses of macrophages to GNPs. In addition to the identified metabolites functioned in distinguished cellular metabolism pathways (Table 6 and Fig 5), a great number of altered glycerophospholipids, fatty acyls, glycerolipids, and sphingolipids was shared or identical in macrophages exposed to GNPs or CD36 ligand (S1–S6 Tables), suggesting that abnormal lipid metabolism is one of the major consequences during the interactions between macrophages and GNPs through the CD36 receptor.

**Table 6:** Selected relevant metabolites to investigate the role of CD36 in GNP-induced macrophage responses.

Metabolite Category	Metabolite	50GNPs vs Ct		100GNPs vs Ct		CD36 vs Ct	
		p-Value	Log FC	p—Value	Log FC	p—Value	Log FC
Pantothenate & CoA Biosynthesis	D-4'-Phosphopantothenate	4.22E-08	-18.71	2.38E-08	-18.27	2.11E-08	-18.13
	Pantetheine	9.13E-07	-17.55	8.63E-07	-17.10	8.45E-07	-16.97
Purine Metabolism	Guanine	2.22E-07	-17.30	1.77E-07	-16.86	1.74E-07	-16.73
	Cholic acid glucuronide	1.12E-07	-18.69	8.40E-08	-18.24	7.71E-08	-18.11
Phenylpropanoid biosynthesis	N1,N5,N10-Triferuloyl spermidine	3.17E-07	-17.88	2.73E-07	-17.44	2.89E-07	-17.31
Argine and proline metabolism	N4-Acetylaminobutanal	7.49E-06	-17.70	7.83E-06	-17.26	7.87E-06	-17.13
	2-deoxyecdysone 22-phosphate	4.39E-08	-17.10	2.14E-08	-16.65	2.02E-08	-16.52
Pyruvate metabolism	(R)-S-Lactoylglutathione	9.64E-09	-16.99	3.40E-09	-16.52	3.32E-09	-16.38
	Lysyl-Isoleucine	3.54E-08	-17.41	1.64E-08	-16.97	1.22E-06	17.55
Glutathione Metabolism	γ-Glu-Cys	8.03E-07	20.86	7.13E-08	20.21	No Change	
	Pyroglutamic acid	2.63E-08	19.26	2.91E-08	20.38	No Change	
	NADPH	6.31E-06	17.75	2.87E-07	17.99	No Change	
	S-Nitroso-L-glutathione	1.46E-07	-17.79	1.13E-07	-17.34	No Change	
Pantothenate & CoA Biosynthesis	Pantothenic Acid	1.83E-07	16.86	3.04E-07	17.44	No Change	
	Adenosine 3',5'-bisphosphate (PAP)	5.56E-08	17.66	5.92E-08	18.44	No Change	
Arachidonic Acide Metabolism	5S-HETE-d8	5.03E-06	-18.32	5.31E-06	-17.87	No Change	
	20-hydroxy-LTE4	7.57E-08	17.15	6.99E-08	17.69	No Change	
Purine Metabolism	Uric acid	2.86E-04	-2.08	2.23E-04	-2.67	No Change	
Riboflavin Metabolism	Riboflavin (Vitamin B2)	6.35E-05	2.50	6.01E-08	17.06	No Change	
	Lumichrome	8.57E-08	17.17	4.63E-08	18.29	No Change	
	Valyl-Gamma-glutamate	1.23E-08	-20.10	4.01E-09	-19.65	No Change	
Skimate/acetate-malonate pathway derived compounds	Isobatatasin I	1.43E-08	-16.99	3.08E-09	-16.55	No Change	
	CPA(18:0/0:0)	3.21E-06	17.07	4.47E-07	17.23	No Change	
	Prolyl-Lysine	9.14E-08	17.27	1.51E-07	18.02	No Change	
Limonene and pinene degradataion Degradation of aromatic compounds	3-Isopropenylpimelyl-CoA	No Change		4.11E-08	18.14	1.27E-07	17.52
Naphthalene degradation Microbial metabolism in diverse environments Degradation of aromatic compounds	2-Naphthoyl-CoA	No Change		3.86E-08	18.16	1.06E-06	17.58
Toluene Degradation	3-Hydroxybenzaldehyde	No Change		No Change		4.40E-09	-20.49
Glutathione Metabolism	Glutathione, oxidized	No Change		No Change		9.15E-06	-19.85
Purine Metabolism	XMP (Xanthosine monophosphate)	No Change		No Change		2.86E-08	-18.30
	Tyrosyl-Aspartate	No Change		No Change		5.13E-07	17.30
	S-Decyl GSH	No Change		No Change		6.11E-06	17.53
	8-Hydroxyadenine	No Change		No Change		1.94E-08	22.34

Note: Ct: control; 50GNPs: 50 μg/mL GNPs; 100GNPs: 100 μg/mL GNPs; FC: fold change.

In living organisms, GSH can be generated through the reduction of GSSG reacting with the coenzyme NADPH, therefore the GSH:GSSG ratio serves as an pivotal bioindicator of normal cellular function with a higher ratio indicating less oxidative stress. Scavenger receptors, particularly the CD36, have been associated with ROS which could trigger the pro-inflammatory signals by modifying proteins, phospholipids, carbohydrates and nucleic acid and mediate the signal transduction leading to disordered cellular physiology [60, 110, 111]. Binding of oxidized phospholipids activates the expression of CD36 and subsequently stimulates the production of ROS, while the deficiency of CD36 reduces the ROS levels, suggesting that CD36 plays a role in regulating the ROS levels [60, 112, 113]. Our data clearly showed that activation of CD36 with SSO markedly reduced the level of GSSG, which could be the consequence of reduced ROS levels caused by deficiency of active CD36. As an intermediate in purine metabolism, XMP is generated from inosine monophosphate (IMP) via the action IMP dehydrogenase and it further undergoes the synthesis of the neucleic acids guanosine monophosphate (GMP) (Fig 5D). A significant down-regulation of XMP induced by activation of CD36 may indicate a disruption in the salvage pathway and de novo synthesis of purines. Further, during the Sphingolipid metabolism, other than being a precursor of ceramide and sphingosine, sphinganine also serves as a substrate of sphingosine kinases to generate sphinganine-1-phosphate (S1P), a bioactive signaling lipid mediator (Fig 5C). A marked reduction in sphinganine following the activation of CD36 implies that CD36 may contribute to mediate S1P signaling transduction. Yet in-depth studies are required to verify the exact relationships between these metabolism pathways and CD36 and whether these relationships contribute to GNPs-induced toxicity.

## Conclusions

In the current study, mouse macrophage responses to GNP exposures including cell viability, cellular uptake, and mitochondrial membrane potential were evaluated. CD36-specific modulators (i.e., ligand and antibody) were also utilized to examine the role of CD36 in mediating the internalization of GNPs and the cytotoxicity induced by GNPs. Further, a metabolomics approach was employed to profile differential responses of macrophages following exposure to GNPs and the role of CD36. Our data demonstrate that (1) GNPs are readily internalized by macrophages and increase the mitochondrial potential following exposure while the pretreatment with CD36 inhibitors (i.e., SSO, CD36 antibody) significantly reduces the internalization of GNPs while blockage of CD36 by use of an antibody inhibited the GNP-induced mitochondrial membrane hyperpolarization; (2) dose- and time-dependent differential metabolomes in macrophages are observed in response to GNP exposure; and (3) specific pathways were determined to be influenced in common by GNP as well as the CD36-specific ligand including glutathione metabolism, pantothenate and CoA biosysnthesis, Sphingolipid metabolism, purine metabolism, and arachidonic acid metabolism. Taken together, our findings provide profound insights of macrophage responses to GNPs and suggest a role of scavenger receptor CD36 in macrophage-GNP interactions. Lastly, our data indicate future directions of investigation regarding GNPs-induced toxicity, immune responses, and potential pathways of response to target for interventions.

## Supporting information

S1 Fig The Raman spectrum of GNPs dispersed in water. (TIFF)

S2 Fig Three-dimensional PCA and Volcano plots showing the dose-dependent metabolite changes in macrophages with or without GNP exposure. (A) PCA and Volcano plots for the comparison of metabolites between control and 50 μg/mL GNPs-exposed macrophages. (B) PCA and Volcano plots for the comparison of metabolites between control and 100 μg/mL GNPs-exposed macrophages. (C) PCA and Volcano plots for the comparison of metabolites between 50 and 100 μg/mL GNPs-exposed macrophages. In the Volcano plot, the horizontal green line represents the significance threshold of p < 0.05, and the vertical green lines indicate the fold change threshold of +2 or -2 folds. The blue squares represent the down-regulated compounds with a fold change less than -2 folds and the red squares represent the up-regulated compounds with a fold change higher than +2 folds.
(TIFF)

S3 Fig PCA and Volcano plots showing the time-course metabolite changes in macrophages exposed to 50 μg/mL GNPs. Macrophages were exposed to 0 or 50 μg/mL GNPs for 1 or 3 h followed by metabolomics analysis. In the Volcano plot, the horizontal green line represents the significance threshold of p < 0.05, and the vertical green lines indicate the fold change threshold of +2 or -2 folds. The blue squares represent the down-regulated compounds with a fold change less than -2 folds and the red squares represent the up-regulated compounds with a fold change higher than +2 folds.
(TIFF)

S4 Fig PCA and Volcano plots showing the CD36-associated metabolite changes in macrophages exposed to CD36 ligand or GNPs. (A) PCA and Volcano plots for the comparison of metabolites between control and CD36 ligand SSO-treated macrophages. (B) PCA and Volcano plots for the comparison of metabolites between CD36 ligand SSO-treated and 50 μg/mL GNP-exposed macrophages. (C) PCA and Volcano plots for the comparison of metabolites between CD36 ligand SSO-treated and 100 μg/mL GNP-exposed macrophages. In the Volcano plot, the horizontal green line represents the significance threshold of p < 0.05, and the vertical green lines indicate the fold change threshold of +2 or -2 folds. The blue squares represent the down-regulated compounds with a fold change less than -2 folds and the red squares represent the up-regulated compounds with a fold change higher than +2 folds.
(TIFF)

S1 Table Complete list of significantly altered metabolite compounds when comparing the control and 50 μg/mL GNP-exposed macrophages (1h time point). (XLSX)

S2 Table Complete list of significantly altered metabolite compounds when comparing the control and 100 μg/mL GNP-exposed macrophages (1h time point). (XLSX)

S3 Table Complete list of significantly altered metabolite compounds when comparing the control and 50 μg/mL GNP-exposed macrophages at 3h time point. (XLSX)

S4 Table Complete list of significantly altered metabolite compounds when comparing the control and 100 μM SSO-treated macrophages (1h time point). (XLSX)

S5 Table Complete list of significantly altered metabolite compounds when comparing the 50 μg/mL GNPs-exposed and 100 μM SSO-treated macrophages (1h time point). (XLSX)

S6 Table Complete list of significantly altered metabolite compounds when comparing the 100 μg/mL GNP-exposed and 100 μM SSO-treated macrophages (1h time point). (XLSX)
