# Supplementary figures and images for: Metabolomic insights of macrophage responses to graphene nanoplatelets: Role of scavenger receptor CD36
Source: PLoS One. 2018 Nov 7;13(11):e0207042. doi: 10.1371/journal.pone.0207042 (PMC6221354; doi:10.1371/journal.pone.0207042)

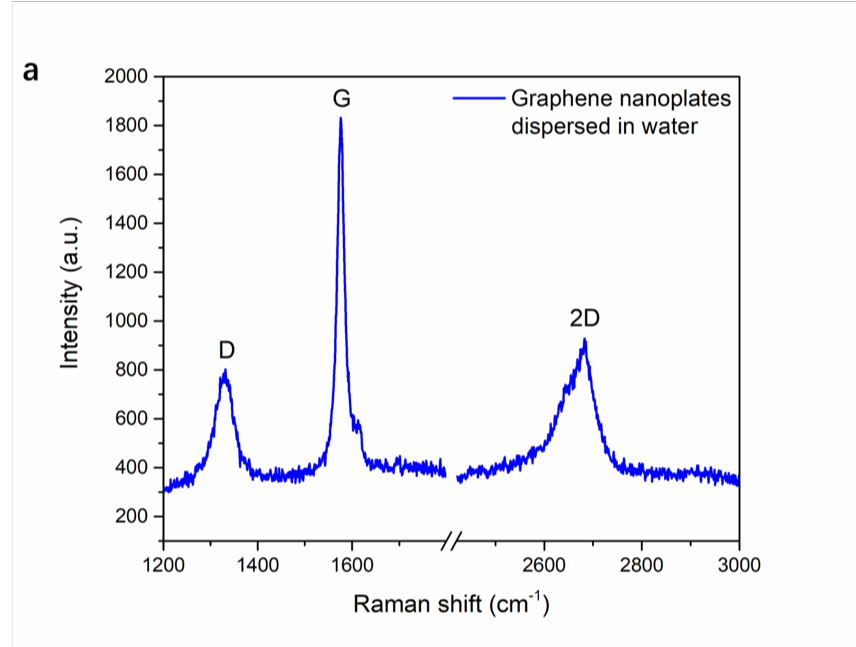

Supplement: S1 Fig — (TIFF) [file pone.0207042.s001.tiff]

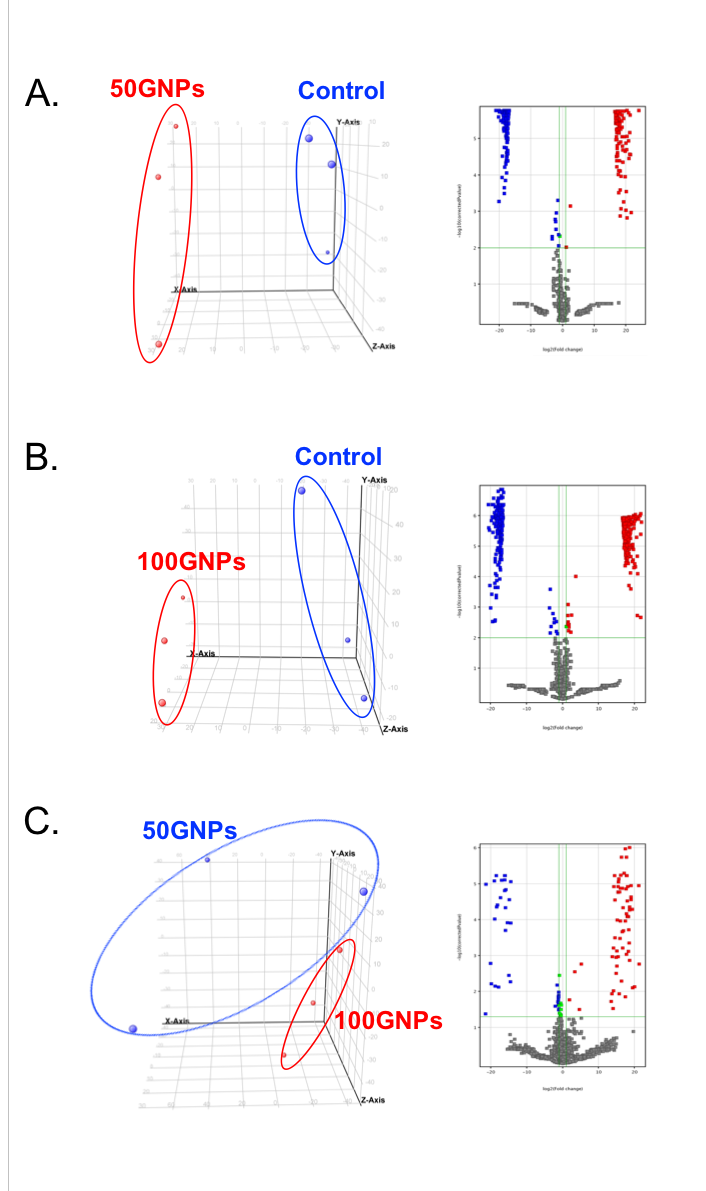

Supplement: S2 Fig — (A) PCA and Volcano plots for the comparison of metabolites between control and 50 μg/mL GNPs-exposed macrophages. (B) PCA and Volcano plots for the comparison of metabolites between control and 100 μg/mL GNPs-exposed macrophages. (C) PCA and Volcano plots for the comparison of metabolites between 50 and 100 μg/mL GNPs-exposed macrophages. In the Volcano plot, the horizontal green line represents the significance threshold of p < 0.05, and the vertical green lines indicate the fold change threshold of +2 or -2 folds. The blue squares represent the down-regulated compounds with a fold change less than -2 folds and the red squares represent the up-regulated compounds with a fold change higher than +2 folds. (TIFF) [file pone.0207042.s002.tiff]

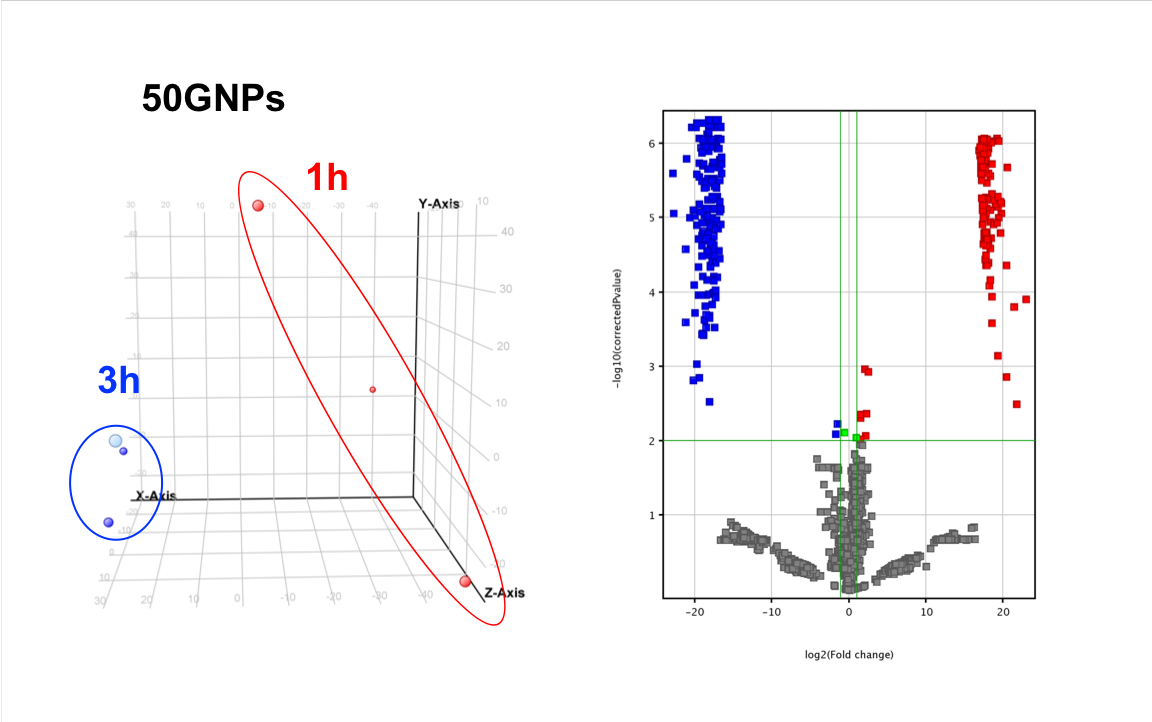

Supplement: S3 Fig — Macrophages were exposed to 0 or 50 μg/mL GNPs for 1 or 3 h followed by metabolomics analysis. In the Volcano plot, the horizontal green line represents the significance threshold of p < 0.05, and the vertical green lines indicate the fold change threshold of +2 or -2 folds. The blue squares represent the down-regulated compounds with a fold change less than -2 folds and the red squares represent the up-regulated compounds with a fold change higher than +2 folds. (TIFF) [file pone.0207042.s003.tiff]

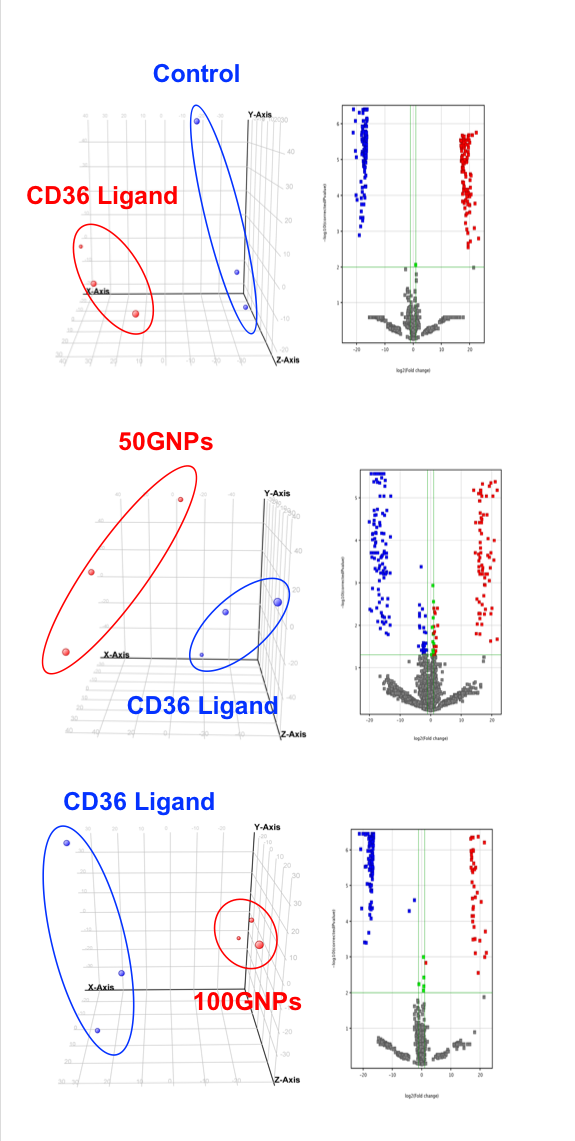

Supplement: S4 Fig — (A) PCA and Volcano plots for the comparison of metabolites between control and CD36 ligand SSO-treated macrophages. (B) PCA and Volcano plots for the comparison of metabolites between CD36 ligand SSO-treated and 50 μg/mL GNP-exposed macrophages. (C) PCA and Volcano plots for the comparison of metabolites between CD36 ligand SSO-treated and 100 μg/mL GNP-exposed macrophages. In the Volcano plot, the horizontal green line represents the significance threshold of p < 0.05, and the vertical green lines indicate the fold change threshold of +2 or -2 folds. The blue squares represent the down-regulated compounds with a fold change less than -2 folds and the red squares represent the up-regulated compounds with a fold change higher than +2 folds. (TIFF) [file pone.0207042.s004.tiff]
